# Genome-wide identification of WD40 transcription factors and their regulation of the MYB-bHLH-WD40 (MBW) complex related to anthocyanin synthesis in Qingke (*Hordeum vulgare* L. var. *nudum* Hook. f.)

**DOI:** 10.1186/s12864-023-09240-5

**Published:** 2023-04-04

**Authors:** Lin Chen, Yongmei Cui, Youhua Yao, Likun An, Yixiong Bai, Xin Li, Xiaohua Yao, Kunlun Wu

**Affiliations:** 1 Academy of Agricultural and Forestry Sciences, Qinghai University, Xining, Qinghai China; 2 Qinghai Key Laboratory of Hulless Barley Genetics and Breeding, Xining, Qinghai China; 3 Qinghai Subcenter of National Hulless Barley Improvement, Xining, Qinghai China; 4 Laboratory for Research and Utilization of Qinghai Tibet Plateau Germplasm Resources, Xining, Qinghai China

**Keywords:** Qingke, MBW complex, *HvWD40-140*, *HvANT1*, *HvANT2*, Anthocyanin

## Abstract

**Background:**

WD40 transcription factors, a large gene family in eukaryotes, are involved in a variety of growth regulation and development pathways. WD40 plays an important role in the formation of MYB-bHLH-WD (MBW) complexes associated with anthocyanin synthesis, but studies of Qingke barley are lacking.

**Results:**

In this study, 164 barley *HvWD40* genes were identified in the barley genome and were analyzed to determine their relevant bioinformatics. The 164 HvWD40 were classified into 11 clusters and 14 subfamilies based on their structural and phylogenetic protein profiles. Co-lineage analysis revealed that there were 43 pairs between barley and rice, and 56 pairs between barley and maize. Gene ontology (GO) enrichment analysis revealed that the molecular function, biological process, and cell composition were enriched. The Kyoto Encyclopedia of Genes and Genomes (KEGG) results showed that the RNA transport pathway was mainly enriched. Based on the identification and analysis of the barley WD40 family and the transcriptome sequencing (RNA-seq) results, we found that *HvWD40-140* (WD40 family; Gene ID: r1G058730), *HvANT1* (MYB family; Gene ID: HORVU7Hr1G034630), and *HvANT2* (bHLH family; Gene ID: HORVU2Hr1G096810) were important components of the MBW complex related to anthocyanin biosynthesis in Qingke, which was verified via quantitative real-time fluorescence polymerase chain reaction (qRT-PCR), subcellular location, yeast two-hybrid (Y2H), and bimolecular fluorescent complimentary (BiFC) and dual-luciferase assay analyses.

**Conclusions:**

In this study, we identified 164 *HvWD40* genes in barley and found that HvnANT1, HvnANT2, and HvWD40-140 can form an MBW complex and regulate the transcriptional activation of the anthocyanin synthesis related structural gene *HvDFR*. The results of this study provide a theoretical basis for further study of the mechanism of *HvWD40-140* in the MBW complex related to anthocyanin synthesis in Qingke.

**Supplementary Information:**

The online version contains supplementary material available at 10.1186/s12864-023-09240-5.

## Background

Qingke (*Hordeum vulgare* L. var. *nudum* Hook. f), a common barley species belonging to the wheat family gramineae, is also called hulless barley or naked barley because its lemma and palea are separated during harvesting [1]. The Qinghai–Tibet Plateau is the main cultivation area of Qingke, and it is distributed in the plateau areas at elevations of 2400–4500 m [1]. It is one of the main food crops for the people in Tibet. Qingke is a highland coarse grain crop with a high nutritional value and is rich in phenolics [2], phytosterols [3], and dietary fiber [4]. Increasing the intake of Qingke can reduce the probability of diseases such as type II diabetes, cardiovascular disease, and cancer [5, 6], therefore, it is a typical whole grain food that is receiving increasing attention as a functional food.

Grains of Qingke have a variety of colors and are classified into five colors: yellow, white, blue, purple, and black. The yellow grains contain procyanidins in the pericarp [7]; the purple and red grains contain anthocyanins in the lemma and pericarp [8]; the black grains contain melanin in the lemma or pericarp [9]; the blue grains contain anthocyanins in the aleurone layer [10]; and the white grains lack biopigments in the outer grain coat, glumes, dextrin layer, and other tissues [11]. Mi et al. [12] found that the average proanthocyanidins and anthocyanidins contents of purple and blue barley are higher than those of black barley. This shows that the difference in color is mainly caused by the accumulation of anthocyanins. Jia et al. [13] showed that the purple grain gene (*HvPre2*) was localized to chromosome 2 H and belonged to the purple barley grain quality trait. Yao et al. [14] found a candidate gene *HvANT1* in the 84.30–86.00 cM region of 7 H, which controls the purple Qingke. Further studies have shown that overexpression of *Ant1* leads to anthocyanin accumulation in the pericarp and dextrin layer of transgenic barley grains [15]. The purple barley gene *Ant2* is located on chromosome 2 H, and the structural gene for anthocyanin biosynthesis in barley is a co-regulated unit in the presence of this gene [8].

WD40 proteins are widely found in eukaryotes and have also been reported in bacteria [16, 17]. The WD40 structural domain has about 40 conserved amino acid residues, usually ending in a tryptophan-aspartic acid (Trp-Asp, WD) at the carboxy (C)-terminus, and are also known as WD40 repeat proteins [18, 19]. WD40 repeats are usually folded into a typical seven-lobe β propeller structure, which surrounds the central chamber, and each blade is composed of four non-parallel strands with a β-chain composition [20]. The WD40 domain itself is not catalytically active, but this propeller structure dictates that it will act as a scaffold or adapter in protein-protein or protein-deoxyribonucleic acid (DNA) interactions and functions [21]. WD40 is mainly found in the plant cytoplasm and is seen as an important regulator in many eukaryotic life processes. It is widely involved in histone modification [22], regulation of phytohormones [23], DNA damage repair [24], and signal transduction [25]. WD40 usually consists of a peptide motif of 44–60 amino acids. It usually consists of a growth hormone (GH) dipeptide (glycine-tryptophan) at the N-terminus and a WD dipeptide (tryptophan-aspartate) at the C-terminus [26]. Recently, the WD40 protein family has been systematically identified in a variety of plant species [25–29]. There are 230 WD40 in *Arabidopsis thaliana*, 200 WD40 in *Oryza sativa*, and 743 WD40 in *Triticum aestivum* [30–32], but no systematic identification and analysis of barley has been reported. The first WD40 protein isolated in plants was *PhAN11* in *Petunia hybrida Vilm* [33]. Humphries et al. [34] showed that the cotton (*Gossypium spp.*) genes *GhTTG1* and *GhTTG3* in the white flowering violet (*Matthiola incana*) Mittg1 mutant are ectopically expressed and can restore some anthocyanin phenotypes. The WD40 protein is not directly involved in target gene promoter-specific recognition but has a more active role in enhancing gene activation. Therefore, it interacts with MYB and bHLH-like transcription factors to form an MBW ternary complex that regulates anthocyanin synthesis [35], which affects anthocyanin accumulation by activating the expression of the structural genes in the anthocyanin synthesis pathway [36–38]. Alan et al. [39] showed that an MBW complex directly regulates the expression of late biosynthetic genes (*LBGs*) in proanthocyanid synthesis in *Arabidopsis thaliana*. Similarly, in Arabidopsis, transparent testa 8 (*TT8*, bHLH), *TT2* (MYB), and transparent testa glabra 1 (*TTG1*, WD40) jointly regulate the biosynthesis of the proanthocyanidins pigments in the grain coat [38]. In grains, the expressions of the structural genes *DFR*, *LDOX*, *BAN*, *TT19*, *TT12*, and *AHA10* are also regulated by MBW [40]. Strygina et al. [41] showed that the pigment accumulation in blue granules in the pasteurized layer is also associated with an MBW complex.

Studies have shown that WD40-like transcription factors affect the synthesis of anthocyanin and proanthocyanidins by interacting with bHLH and MYB transcription factors to form complexes [38]. In this study, the WD40 family was identified in barley genome, using bioinformatics methods of determining the physicochemical properties, conserved structural domains, protein conserved motifs, phylogenetic analysis, Gene Ontology (GO) and Kyoto Encyclopedia of Genes and Genomes (KEGG) analysis of barley WD40 transcription factors were performed. In addition, we obtained an MBW complex composed of *HvWD40-140* (WD40), *HvANT1* (MYB), and *HvANT2* (bHLH) via transcriptome sequencing (RNA-seq) [1], and then, we verified their expression patterns, subcellular localization, protein-protein interactions and dual-luciferase assay. The results of this study further elucidate the function of the WD40 protein and its deep regulatory mechanism and reveal the anthocyanin synthesis network.

## Results

### Identification of WD40 family genes in barley

**Fig. 1:**
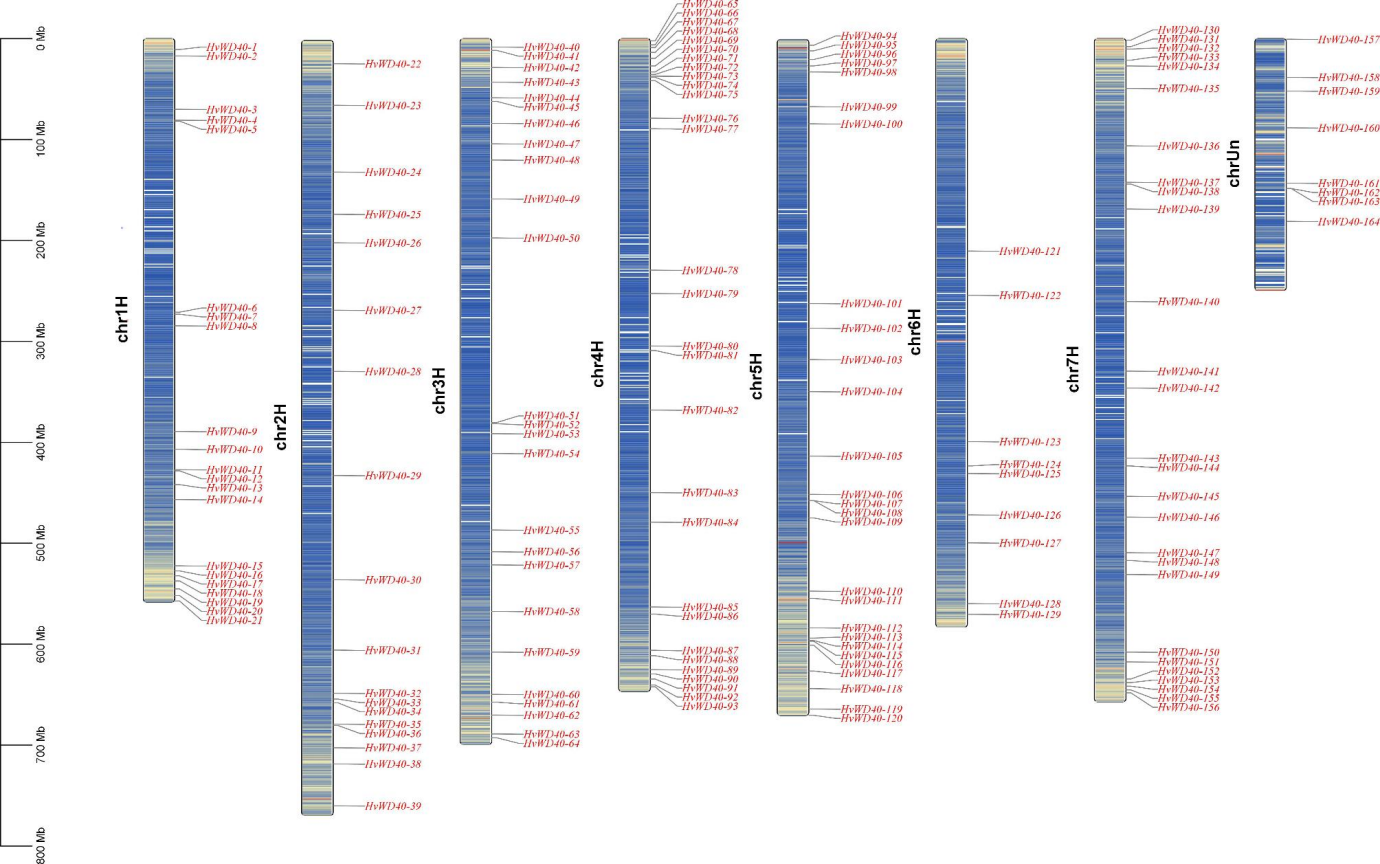
Chromosome mapping of 164 *HvWD40s*. The scale bar on the left indicates the length (Mb) of the barley chromosome. The chromosome number is shown on the left side of each chromosome, and the gene number is shown on the right side of each chromosome

A total of 164 *HvWD40s* were identified based on genome-wide analysis and were named *HvWD40-1* to *HvWD40-164* based on their locations on the barley genome (Fig. 1). A total of 155 *HvWD40* genes were unevenly distributed on seven chromosomes; and the chromosomal locations of eight genes were indeterminate. Chromosome 4 H had the highest number of *HvWD40*s (29), followed by 3 H, 5 H, and 7 H with 25, 27, and 27, respectively, and only 9 were located on chromosome 6 H (Additional file 1: Table S1).

The subcellular localization prediction results for the 164 HvWD40s revealed that 67 were located in chloroplasts, 55 in the nucleus, 30 in the cytoplasm, 2 in the endoplasmic reticulum, 1 in the vesicle, 2 in the cytoskeleton, 1 in the endosome, 1 in the plasma membrane, and 5 in the mitochondria. WD40 proteins most likely involved in the transcription factors since they were mostly found in the nucleus. HvWD40s varied largely in length and physicochemical properties. The lengths of the amino acids ranged from 67 (HvWD40-34) to 3288 (HvWD40-114). The molecular weights (MWs) ranged from 7391.41 (HvWD40-34) to 366074.54 kDa (HvWD40-114). The isoelectric point (pIs) ranged from 4.24 (HvWD40-155) to 11.43 (HvWD40-99). The protein instability indices (II) ranged from 22.25 (HvWD40-25) to 64.22 (HvWD40-11), and the aliphatic indices ranged from 47.14 (HvWD40-126) to 97.41 (HvWD40-12). The average protein hydrophilicity (GRAVY) ranged from 52.48 (HvWD40-111) to 0.474 (HvWD40-3). HvWD40-101 and HvWD40-129 had signal peptides, the remaining proteins had no signal peptides, and none of the proteins had transmembrane structures (Additional file 2: Table S2).

### Phylogenetic tree construction and classification of barley WD40 proteins

**Fig. 2:**
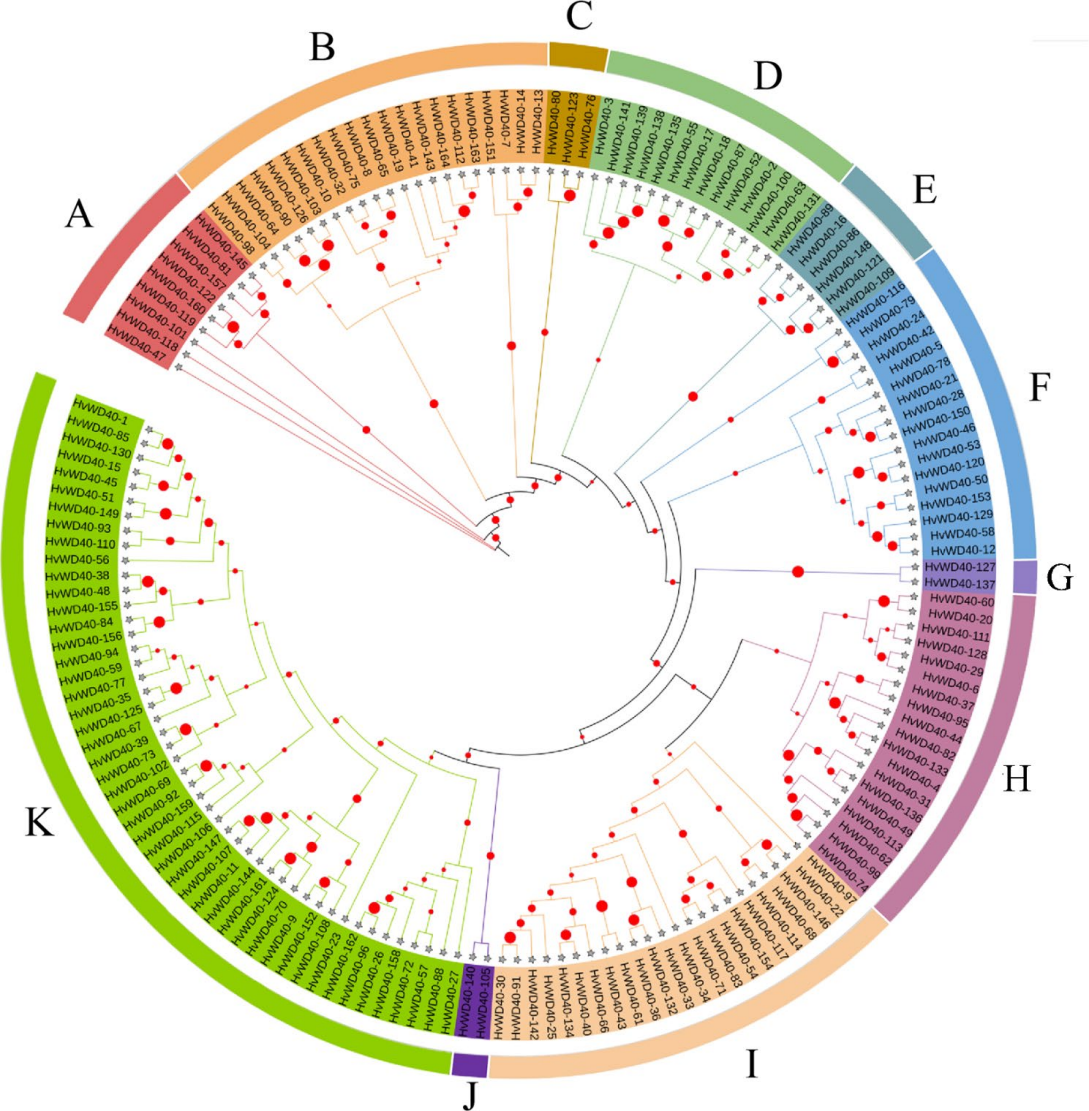
Phylogenetic classification of HvWD40 proteins. Different colors areas denote clusters A–K, and the size of the red dots indicates the bootstrap. The phylogenetic tree was constructed using MEGA 7.0 and the neighbor joining (NJ) method with 1,000 bootstrap replications

To further analyze the affinities of the barley WD40 families, 164 protein sequences identified as HvWD40s were used to construct a phylogenetic tree (Fig. 2). It was divided into 11 groups (clusters A–K) containing 9, 21, 3, 14, 6, 17, 2, 19, 23, 2, and 48 members. The 164 HvWD40s were classified into 14 subfamilies based on their domain composition (Table 1), of which 129 HvWD40s with only the WD40 domain were classified as subfamily 1; eight HvWD40 containing either the WD40 domain and the LisH domain or the WD40 domain and both LisH and CTLH domain were classified as subfamily 2; six HvWD40 containing the WD40 domain and either UTP12 or UTP15 were classified as subfamily 3; four HvWD40 containing the WD40 domain and the beach domain were classified as subfamily 4; three HvWD40 containing the WD40 domain, the Coatomer_WDA D (Coatomer WD related region) domain, and the COPI_C (Coatomer alpha subunit C-terminal) domain were classified as subfamily 5; and three HvWD40 containing the WD40 domain and F-BOX/U-BOX were classified as subfamily 7. Subfamily 8, subfamily 10, and subfamily 11 contained two HvWD40, containing the WD40 domain with the Pkinase domain, the WD40 domain with the BING4CT domain, and the WD40 domain with the BOPINT domain, respectively. Subfamily 6, subfamily 9, subfamily 12, and subfamily 13 all contained only one HvWD40 protein, containing the WD40 domain with the NLE domain, the WD40 domain with the CAFIC_H4-bd domain, the WD40 domain with the PRP4 domain, and the WD40 domain with the Hira domain, respectively. Four HvWD40 containing the WD40 domain and other domains were classified as subfamily 14.

Then, we identified a total of nine motifs, named motif 1 to motif 9 (Additional file 6: Fig. S1B). The HvWD40 protein motifs located in the same group were similar in class and order, whereas the number of motifs in the different groups varied considerably. Motifs 1, 2, 3, 4 (the most distributed motif), 5, 6, 7, 8, and 9 (the least distributed motif) contained 140, 147, 144, 157, 139, 126, 106, 123, and 3 HvWD40 proteins, respectively.

**Table 1:** Domain composition and number of members of the 14 subfamilies of 164 HvWD40s

Subfamily	Domain composition	Number of members
Subfamily 1	Only WD40 domain	129
Subfamily 2	WD40 domain and LisH domain/LisH + CTLH	8
Subfamily 3	WD40 domain and UTP12 or UTP15	3
Subfamily 4	WD40 domain and Beach domain	4
Subfamily 5	WD40 domain and WDAD or WDAD + COPIC	3
Subfamily 6	WD40 domain and NLE domain N terminal	1
Subfamily 7	WD40 domain and U-box or F-BoX	3
Subfamily 8	WD40 domain and Pkinase domain	2
Subfamily 9	WD40 domain and CAFIC_H4-bd domain	1
Subfamily 10	WD40 domain and BING4CT domain	2
Subfamily 11	WD40 domain and BOPINT domain	2
Subfamily 12	WD40 domain and PRP4 domain	1
Subfamily 13	WD40 domain and Hira domain	1
Subfamily 14	WD40 domian and domains with unknown function	4

### Diversity of *HvWD40* gene structures and main promoters cis‑element regulator analysis

The gene structure results showed that the number of exons and introns in the *HvWD40s* gene family varied considerably (Additional file 6: Fig. S1C). Seventeen HvWD40 genes had only one exon and no introns. One hundred HvWD40 genes contained 1–9 exon, 59 HvWD40 genes contained 10–20 exons, and only 5 HvWD40 genes contained more than 20 exons. The cis-element analysis of the promoter regions of the 164 HvWD40 genes revealed that they contained elements such as gibberellin (P-box), growth hormone (AuxRE), and abscisic acid (ABRE), which are related to the biotic stress response, light regulation, and response to plant physiological regulation. They also contained cis-elements for abiotic stresses and immune responses, such as MYB, MYC binding sites, anaerobic (ARE), low temperature (LTR), and defense (TC-rich repeats) (Additional file 7: Fig. S2). Thus, *HvWD40* may be associated with the regulation of the expression of genes related to plant physiology, abiotic stress, and immune response.

### Genome-wide covariance analysis of barley with rice and maize

**Fig. 3:**
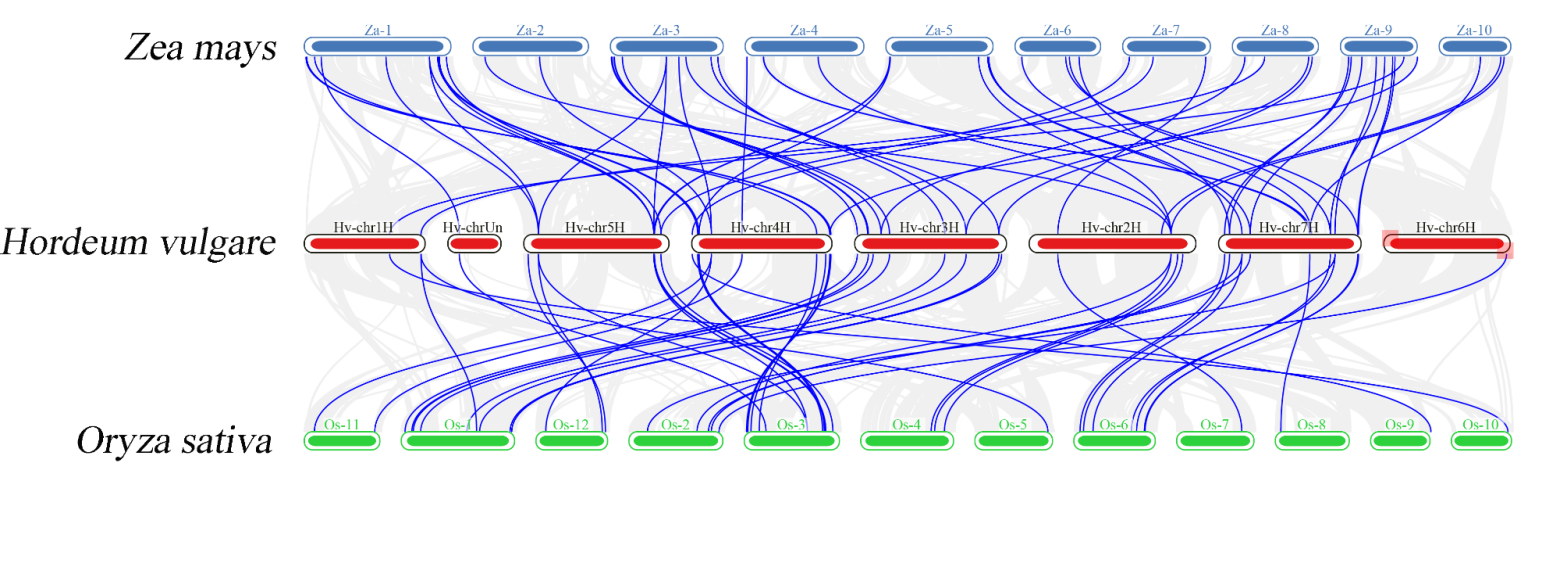
Collinear relationships between barley and rice and maize. The identified collinear genes are linked by blue lines

To further investigate the potential evolution of the *HvWD40* gene family, genome-wide covariance analysis was performed between barley and rice and maize (Fig. 3). A total of 43 pairs of genes in barley and rice were co-linked; and a total of 56 pairs of genes in barley and maize were co-linked (Additional file 3: Table S3). As can be seen from the above results, the number of collinear gene pairs between barley and rice was much greater than that between barley and maize. These segmentally duplicated *WD40* genes have maintained a replicative chain throughout their long evolutionary history, and they may have similar functions.

### The expression of *HvWD40* gene in RNA-seq

**Fig. 4:**
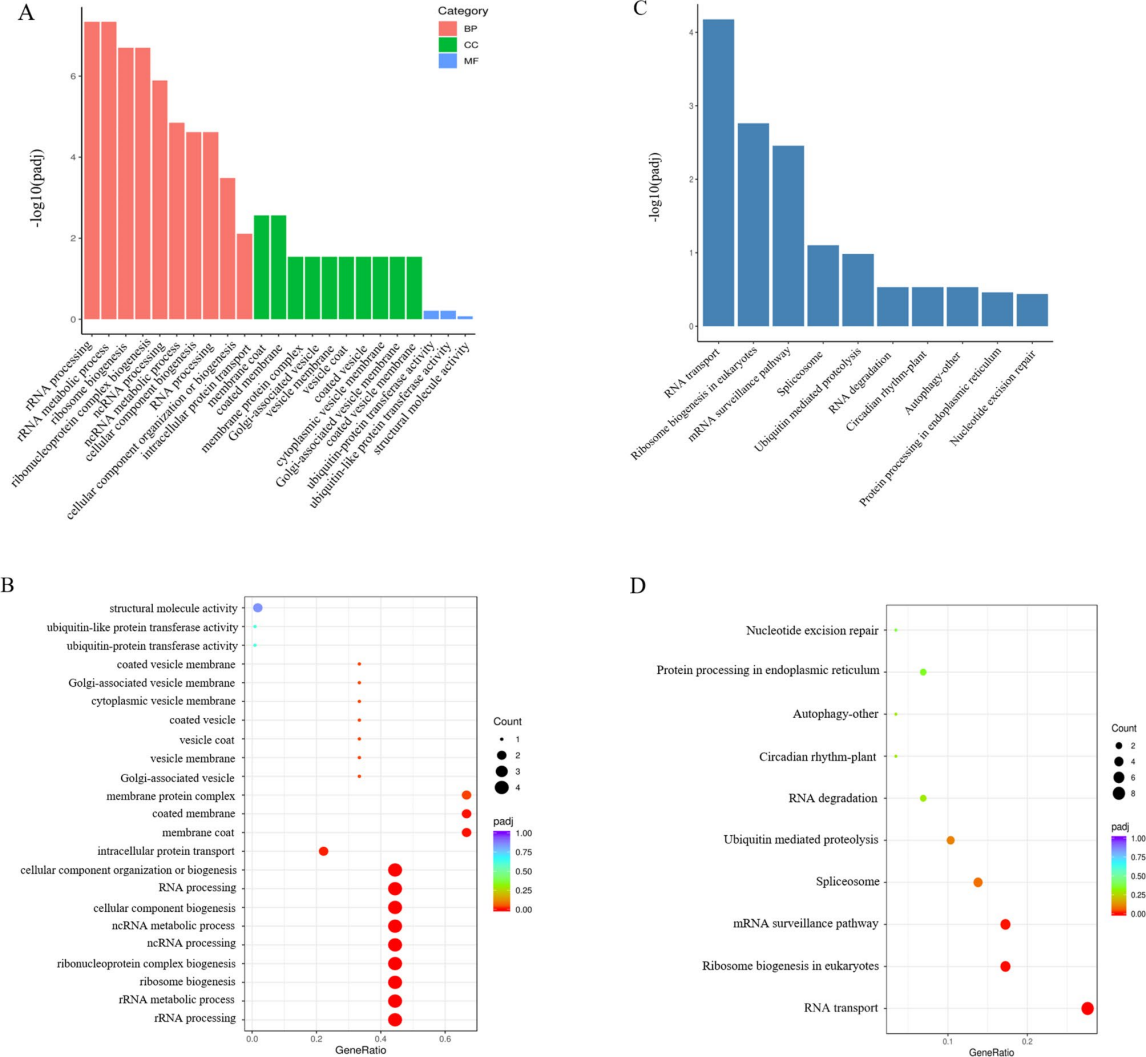
Go and KEGG enrichment analysis of 164 *HvWD40* genes. **A** GO enrichment bar graph. The first 23 terms of the significance analysis were selected for display. The Y axis represents the p-value, and the X axis represents the GO term. BP biological process; CC cell composition, and MF molecular function. **B** GO enrichment bubble graph. The Y axis is the GO term, and the X axis is the enrichment factor. **C** KEGG enrichment bar graph. The first 10 pathways of the significance analysis were selected for display. The Y axis represents the p-value and the X axis represents the top 10 pathways [42–44]. **D** KEGG enrichment bubble graph. The Y axis is the enrichment pathway and the X axis is the enrichment factors. The bubble color represents the p-value, the redder the color is, the smaller the p-value is, the higher the enrichment degree is, and the size of the bubble indicates the quantity [42–44]

**Fig. 5:**
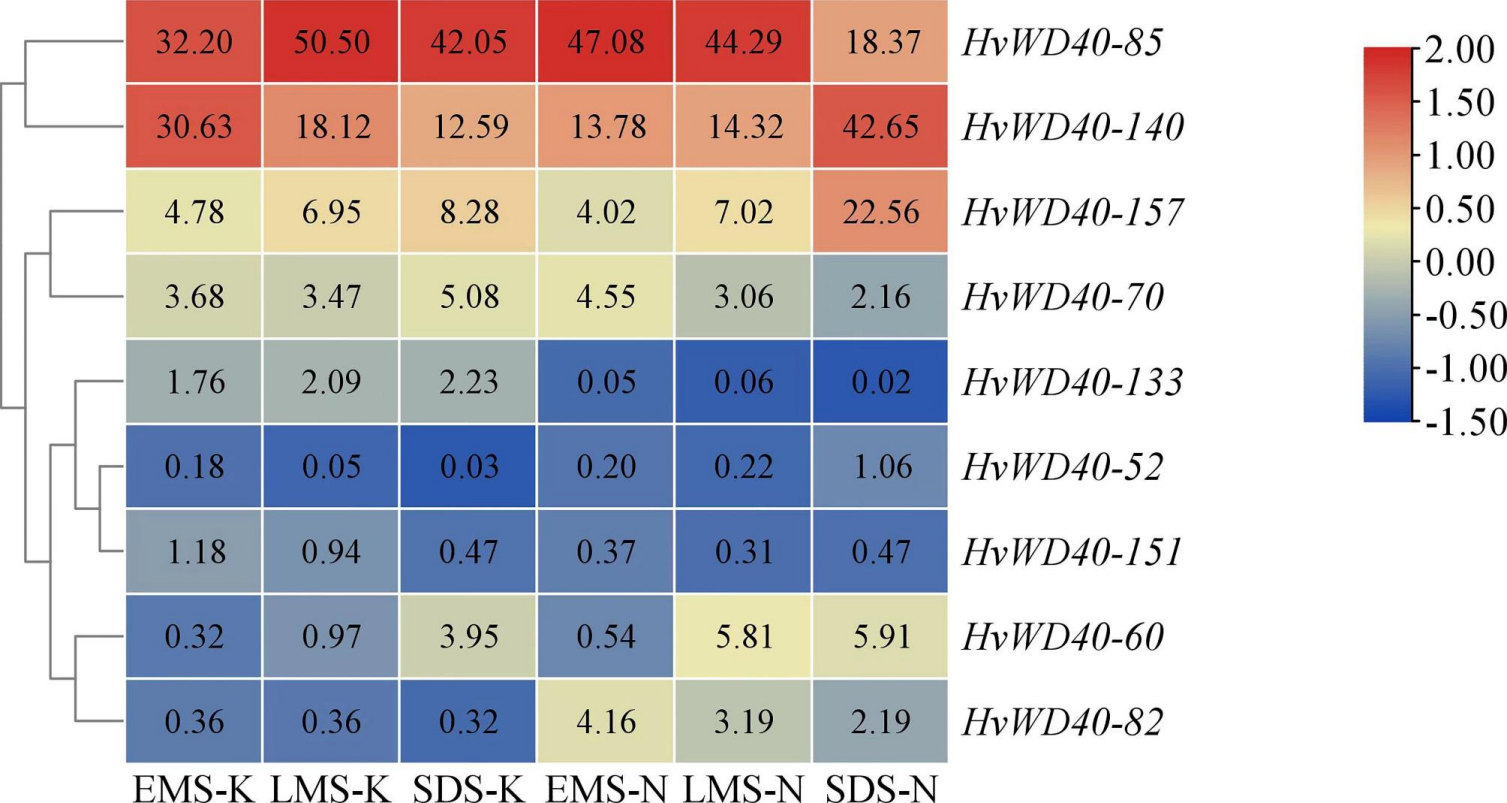
Expression profiling of differentially expressed *HvWD40* genes. EMS-K: early milk stage of Kunlun 10; LMS-K: late milk stage of Kunlun 10; SDS-K: soft dough stage of Kunlun 10; EMS-N: early milk stage of Nierumuzha; LMS-N: late milk stage of Nierumuzha; and SDS-N: soft dough stage of Nierumuzha. The heatmap was generated based on the RNA-seq data and was drawn using the TBtools program. The different cell colors correspond to the log10 magnitude of the difference in the expression level [log10 (fold change values + 1)]. A redder cell color indicate upregulation, while bluer cells color indicate downregulation

To understand the function of 164 *HvWD40s*, GO and KEGG analyses were performed. Significant GO enrichment was determined at a threshold of *P* ≤ 0.05. We obtained 45 enriched types, containing 27 biological processes, 30 cellular compositions, and 3 molecular functions (Additional file 4: Table S4). The results revealed that the *HvWD40* family genes were mainly enriched in biological processes, including ribosomal ribonucleic acid (rRNA) processing, rRNA metabolic process, and ribosome biogenesis (Fig. 4A and B). The KEGG analysis results revealed that RNA transport, ribosomal biogenesis in eukaryotes, mRNA monitoring pathway, spliceosome, ubiquitin-mediated proteolysis, RNA degradation, circadian rhythm-plant autophagy, protein processing in endoplasmic reticulum, nucleotide excise-repair, and other pathways were significantly enriched (Fig. 4 C and 4D) [42–44]. Eight of these genes were contained in the RNA transport pathway, which was the pathway with the highest number of genes (Additional file 4: Table S4). In order to further understand the role of these genes in the anthocyanin synthesis of the purple Qingke, we analyzed the RNA-seq results of 164 *HvWD40s* in the early milk stage, late milk stage, and soft dough stage of white-grain Kunlun 10 and purple-grain Nierumuzha. We found that 164 *HvWD40* genes were expressed in the different stages of the different varieties, and the expression levels of most of the *HvWD40s* were higher in Nierumuzha than in Kunlun 10 (Additional file 8, Fig. S3). The expression levels of nine genes were different for the two cultivars (*P* < 0.05; Fig. 5). In addition, we found that the expression of *HvWD40-140* increased with the development of Nierumuzha grains, while the opposite occurred in Kunlun 10.

### Screening of MWB complex (MYB, bHLH, and WD40) genes related to anthocyanin synthesis

**Fig. 6:**
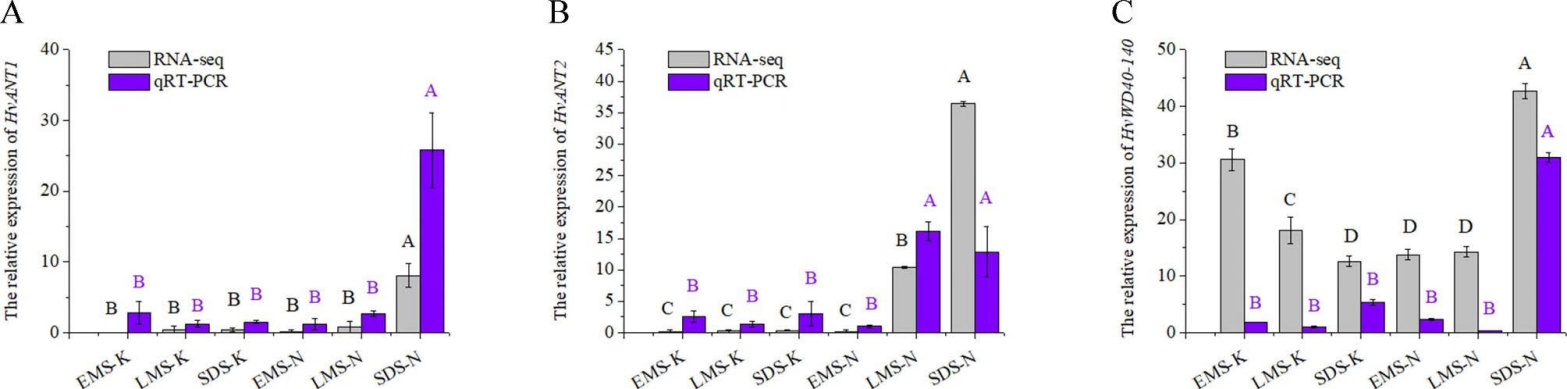
Relative expressions of *HvANT1*, *HvANT2*, and *HvWD40-140* in Kunlun 10 and Nierumuzha. The *TC139057* were used as internal. The error line represents the standard deviation (n = 3). Significant differences were determined via one-way analysis of variance, and the different capital letters indicate highly significant differences (*P* < 0.01). EMS-K: early milk stage of Kunlun 10; LMS-K: late milk stage of Kunlun 10; SDS-K: soft dough stage of Kunlun 10; EMS-N: early milk stage of Nierumuzha; LMS-N: late milk stage of Nierumuzha; and SDS-N: soft dough stage of Nierumuzha. Gray indicates RNA-seq, and purple indicates qRT-PCR.

**Fig. 7:**
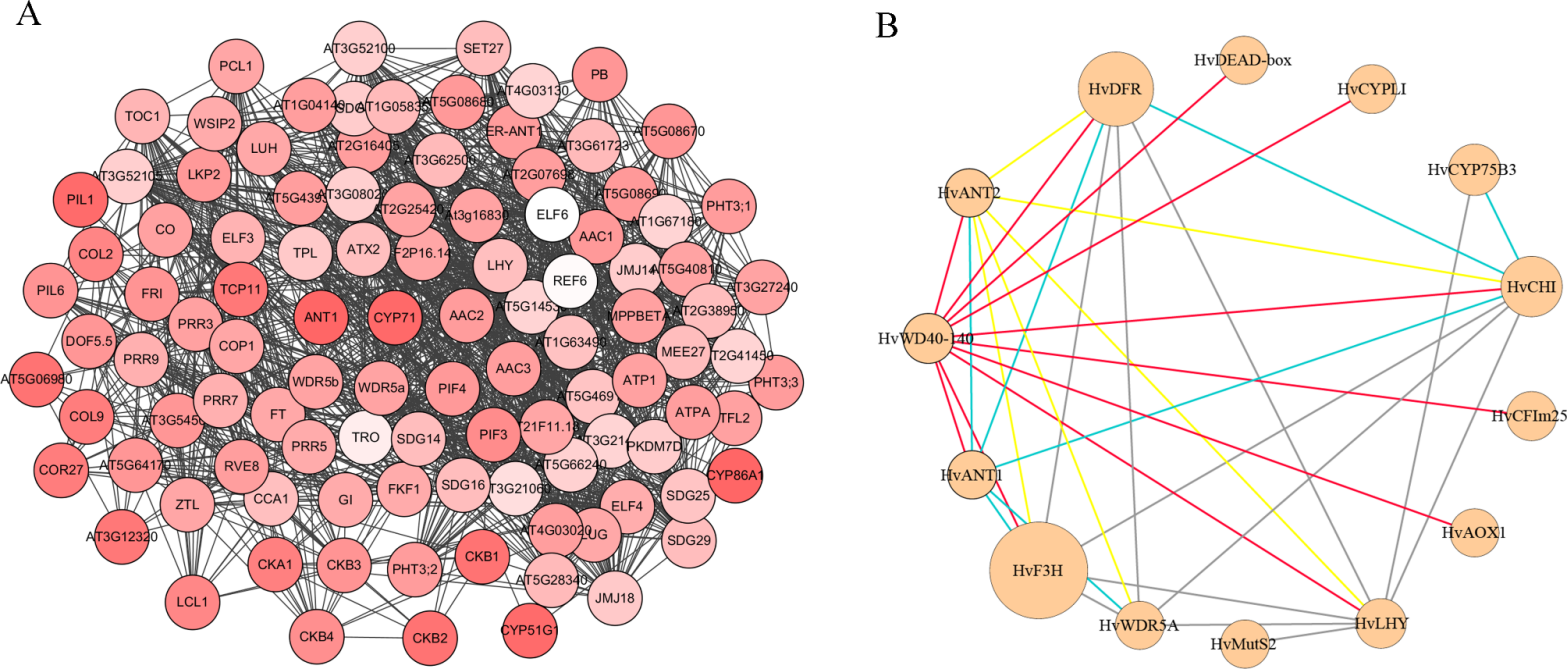
Prediction of protein interactions. **A** STRING online protein interaction prediction. The depth of the color indicates the degree score **B** Protein interaction prediction based on RNA-seq using cytoscape. The size of the circle represented the expression level of genes in the soft dough stage formed by the grain color of the Nierumuzha, the red line represents proteins that interact with HvWD40-140, the blue line represents proteins that interact with HvANT1 and the yellow line represents proteins that interact with HvANT2, and the gray line shows other proteins interacting

To further investigate the anthocyanin synthesis regulatory mechanism of the *HvWD40* family in Qingke, we found that three transcription factors, *HvANT1* (HORVU7Hr1G034630), *HvANT2* (HORVU2Hr1G096810), and *HvWD40-140* (HORVU7Hr1G058730), were highly significantly differentially expressed in the soft dough stage of purple Qingke based on the RAN-seq results (*P* < 0.01). The RNA-seq and qRT-PCR results revealed that these three genes had similar expression patterns, that is, decreasing or unchanged expression in Kunlun 10 and increasing expression in Nierumuzha (Fig. 6). The differences in the *HvANT1* expression during all three periods for Kunlun 10 were not significant. The expression of the *HvANT1* gene in the soft dough stage of Nierumuzha was significantly higher than that of Kunlun 10 (*P* < 0.01; Fig. 6A). The difference in the expression of the *HvANT2* gene in Kunlun 10 was not significant, but it was highly significant in Nierumuzha (*P* < 0.01), and the expression in both the late milk stage and the soft dough stage was highly significantly higher in Nierumuzha than in Kunlun 10 (*P* < 0.01). The expression of the *HvWD40-140* gene was reduced in Kunlun 10 (*P* < 0.01), while it decreased and then increased in Nierumuzha, with a highly significant difference (*P* < 0.01). In addition, we found that these three proteins interacted with each other and with structural genes associated with anthocyanin synthesis. The homologous proteins AtANT1, AtAAC2, and AtCYP71 of HvANT1, HvANT2, and HvWD40-140 interacted with each other in the Arabidopsis interaction model in STRING (Fig. 7A). We found that HvANT1, HvANT2, and HvWD40-140 interacted with structural genes associated with anthocyanin synthesis based on the protein-protein interaction (PPI) results of the RNA-seq (Fig. 7B).

### HvANT1, HvANT2, and HvWD40-140 subcellular locations

**Fig. 8:**
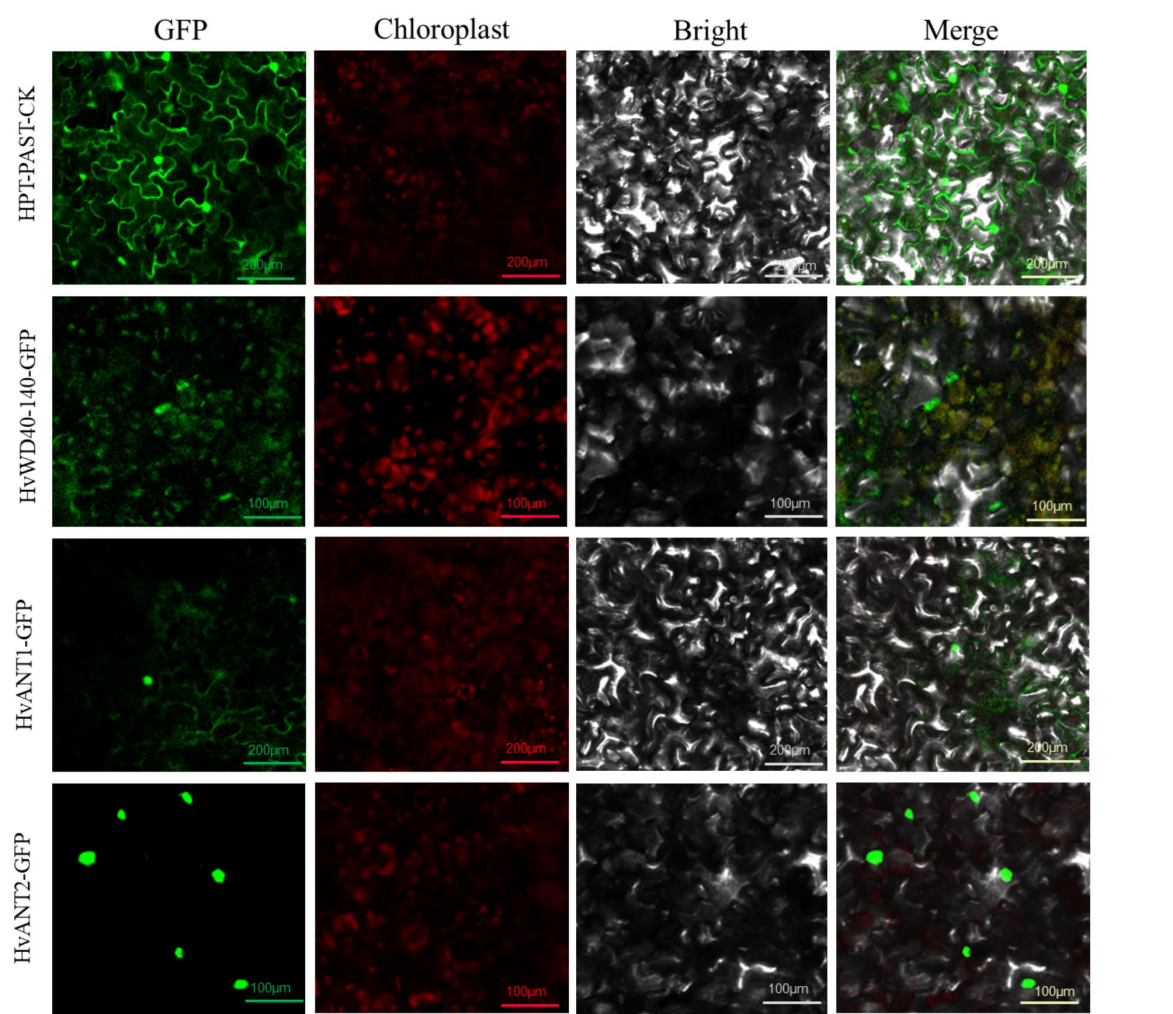
Subcellular locations of HvANT1, HvANT2, and HvCYP71. HvANT1-GFP was located in the nucleus and cell membrane, HvANT2-GFP was located in the nucleus, HvWD40-140 was located in the chloroplast. Images of GFP, chlorophyll autofluorescence, bright field, and GFP merged with bright field (Merge) are shown. The scale bars are 100 or 200 μm

To understand the protein characteristics of HvANT1, HvANT2, and HvWD40-140, the subcellular localization of this proteins were detected. *Nicotiana benthamiana* leaves expressing the green fluorescent protein (GFP) protein were analyzed using a confocal microscope. HvANT1-GFP was expressed in the nucleus and cell membrane, HvANT2-GFP was expressed in the nucleus, and HvWD40-140-GFP was expressed in the chloroplast. Therefore, HvANT1 was localized in the nucleus and cell membrane, HvANT2 was localized in the nucleus, and HvWD40-140 was localized in the chloroplast (Fig. 8).

### HvANT1 and HvANT2 interacted with HvWD40-140 protein

**Fig. 9:**
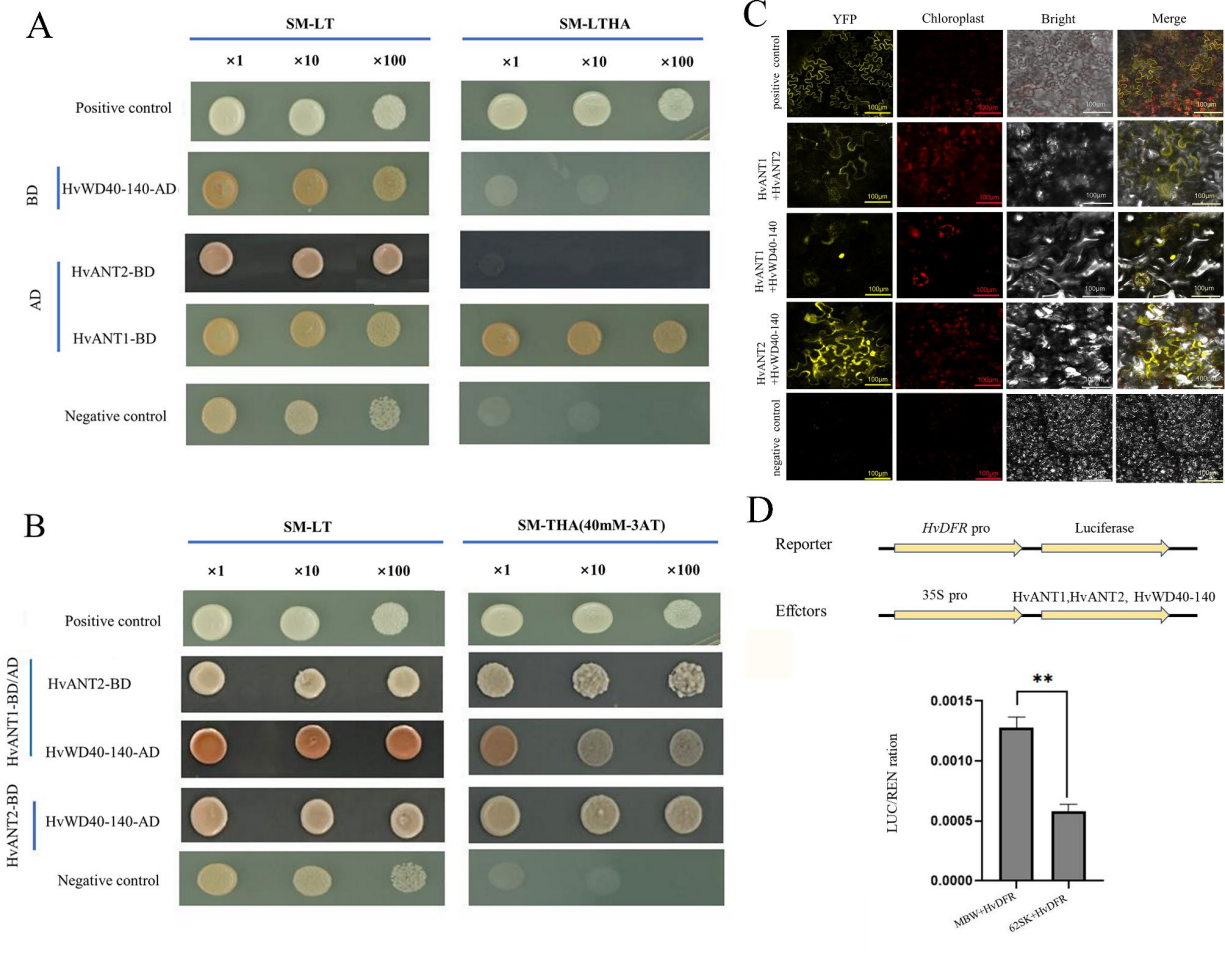
HvANT1 and HvANT2 interact with HvWD40-140 proteins and form MBW complexes with three transcription factors to verify *HvDFR* promoter transcriptional activation **A** Validation of protein self-activation of HvANT1, HvANT2, and HvWD40-140. **B** Validation of interaction between HvANT1, HvANT2, and HvWD40-140 were analyzed via yeast two-hybrid assay. -LT denotes SD/-Leu-Trp, and-THA denotes SD/-Trp-His-Ade. The yeasts were grown in the absence of Leu and Trp (SD-LT) for selection of co-transformed cells. east cells were incubated to OD600 = 1 and diluted 1-, 10- or 100-fold for assay. The assay of the auto-activation of and interactions between the tested genes was conducted in the absence of Trp, His, and Ade (SM-THA). BD-53 + AD-T7 is a positive control, BD-53 + AD-T is a negative control. **C** Validation of interaction between HvANT1, HvANT2, and HvWD40-140 were analyzed via BiFC. HvANT1 was fused with the N-terminal and C-terminal fragments of yellow fluorescent protein (YFP), HvANT2 as fused with the N-terminal fragment, and HvWD40-140 as fused with the C-terminal fragment. It was found that HvnANT1 and HvnANT2 interacted with HvWD40-140 to form biomolecular fluorescent complexes. Images of YFP, chlorophyll autofluorescence, bright field, and YFP merged with bright field (Merge) are shown. The scale bar indicates 100 μm. **D** HvANT1-HvANT2-HvWD40-140 transcription factor complexes activate *HvDFR* promoter. Effector constructs and *HvDFR* promoter-driven reporter gene construct and promoter activity of *HvDFR* in tobacco leave protoplasts is activated by the co-expression of *HvANT1* with *HvANT2* and the *HvWD40-140* genes. Promoter activities are expressed as the Firefly/Renilla luciferase activity ratio. The data are from three biological replicates and are expressed as the mean SD.

Using the yeast two-hybrid and bimolecular fluorescent complimentary technique, the interactions between the three types of transcription factors were explored in vitro. The Y2H results showed that HvANT2-BD and HvWD40-140-AD did not have self-activation, whereas HvANT1-BD had self-activation (Fig. 9A). The drug-selective marker 3-amino-1,2, 4-triazole (3-AT) was used to inhibit the self-activation. HvANT1-BD did not grow on yeast medium synthetic defined Trp-Leu (SD-TL) when 40 mM 3-AT was added. Therefore, we performed protein interaction verification in self defined Trp-His-Ade (SD-THA) medium supplemented with 40 mM 3-AT, and the results showed that HvANT1 and HvANT2 interacted with HvWD40-140 (Fig. 9B). In addition, we used the bimolecular fluorescence complementation technique to validate the above results. HvANT1 and HvANT2 were expressed in the cell membrane, as well as HvANT2 and HvWD40-140 were expressed in the cell membrane; and HvANT1 and HvWD40-140 were expressed in the nucleus (Fig. 9C). Thus, both yeast two-hybrid assay and the bimolecular fluorescence complementary technique revealed that HvANT1 and HvANT2 interacted with HvWD40-140. In addition, we conducted a dual-luciferase assay to measure the promoter activation in tobacco (*Nicotiana benthamiana*) leaves, and we found that the Firefly/Renilla (LUC/REN) ratio was significantly higher (2.21 times) than that of *HvDFR* when *HvANT1*, *HvANT2*, and *HvWD40-140* were co-expressed (Fig. 9D). These data suggest that the co-expression of *HvANT1*, *HvANT2*, and *HvWD40-140* activates the activity of the *HvDFR* promoter and can form MBW complexes to promote *HvDFR* express.

## Discussion

Previous studies have shown that WD40 generally does not have a catalytic activation function, but rather it functions as an effector with another of the MYB and bHLH proteins, enhancing the activation of the MBW (MYB-bHLH-WD40) complex by forming a ternary complex without directly participating in the recognition of the target gene promoters [25, 40, 45]. In the MBW complex, the WD40 protein is usually located at the center of the ternary structure and may protect the MBW complex by preventing other transcriptional regulators from binding to the MYB or bHLH [45, 46]. In plants, WD40 repeat proteins are widely involved in a variety of cellular processes, including signal transduction, cell wall formation, chromosome remodeling and histone modification, proteasomal degradation, and microtubule tissue assembly [25]. Numerous studies have shown that the MBW complex plays an important role in the anthocyanin biosynthetic pathway [38], but little has been reported about the regulation of the synthesis of anthocyanins in barley. Therefore, in this study, the barley WD40 gene family was identified and then analyzed at the barley genome-wide level. Hu et al. [30] identified 743 WD40 family members in wheat (*Triticum aestivum*) and reported that the highest number of gene members contained 1–9 exons (312 genes), with 7–14 exons in about half of the *TaWD40s*. In this study, in barley, the number containing 1–9 exon genes (100 genes) were the largest, while 77 *HvWD40s* genes (about half of the *HvWD40* family) contained 7–14 exons, which was similar to that of wheat. This indicates that the gene structure is highly conserved. In rice the WD40 proteins were also divided into 11 subfamilies based on their structural domain composition [32]. The subfamily clustering analysis conducted in this study revealed that the 164 barley WD40 proteins were also divided into 11 groups. Conservative domain analysis showed that the 164 barley HvWD40 proteins were clustered into 14 subfamilies, and all of the HvWD40 protein members in each subfamily had a WD40 domain. Subfamily 1 was the most abundant and only contained the WD40 domain, while the other subfamilies contained domains such as the NLE, protein kinase, and LisH domains. The WD40s proteins of wheat, rice, and potato (*Solanum Tuberosum*) [30, 32, 47] species contain WD40 domains, and the WD40 protein in the most abundant subfamily contains only the WD40 domain, while the other subfamilies contain only the WD40 domain. Domains such as the NLE, F-BOX, U-BOX, and LisH domains are also included. These previous results are similar to those of this study, indicating that the WD40 domain has remained relatively unchanged during evolution, which is consistent with the characteristics of the WD40 protein. These results confirm that most of the WD40 proteins with the same domain cluster together, and thus can also adapt to more biological processes or more precisely regulate a biological process. Temporal and spatial expression profile analysis revealed that many of the *HvWD40* genes were differentially expressed in the different varieties during the key period of grain color formation, indicating that *HvWD40* may be involved in anthocyanin accumulation. Among the nine differentially expressed genes, the *HvWD40-140* gene expression increased in Nierumuzha and decreased in Kunlun 10, suggesting that this gene may be involved in the synthesis of anthocyanin in Qingke grain.

The results of Zhou et al. [15] showed that barley *Ant1* and *Ant2* play an important role in the regulation of purple barley grains. However, there are no relevant reports on which *WD40*, *ANT1*, and *ANT2* combine to form an MBW complex. We also located the *ANT1* gene in the positioning segment during the color localization of Qingke purple grains [14]. Therefore, this study aimed to explore the function of the MBW complex in the anthocyanin synthesis process in Qingke, and analysis was conducted based on the biological information about the WD40 gene family and the results of the RNA-seq. *HvWD40-140*, *HvANT1*, and *HvANT2*, which may play an important role in MBW complex reactivation, were obtained. At present, there are many reports that WD40 can form an MBW complex with MYB and bHLH to participate in anthocyanin synthesis in plants. For example, in cherry (*Prunus avium*), *PavMYB10.1a*, *PavbHLH*, and *PavWD40* interact with each other, and a terplex complex may be formed to regulate the expression of the downstream structural genes *PavANS* and *PavUFGT* [48]. In potato, when three MYB genes (*StAN1*, *StMYBA1*, and *StMYB113*) are co-expressed with the *bHLH* gene, anthocyanin synthesis in tobacco can be regulated [49]. In *Medicago truncatula*, *MtTT8*, *MtWD40-1*, and the MYB transcription factors *MtPAR* or *MtLAP1* form a terplex complex regulatory structural gene *MtANS* and the *MtANR* gene transcription, thus regulating the synthesis of anthocyanins and proanthocyanidins [37]. The *ZmPAC1* gene in maize can encode the WD40 protein, which is similar to *AN11* and *TTG1*, and can regulate anthocyanin synthesis when co-expressed with *ZmR1* (bHLH) and *ZmC1* (MYB) [50]. These results suggest that WD40 can form ternary complexes with MYB and bHLH to regulate anthocyanin synthesis. In Arabidopsis, *TT2*, *TT8*, and *TTG1* can form ternary complexes, and *TTG1* regulates the *BAN* expression, mainly by influencing the *TT8* function [40]. *TTG1* and *GL1* are lost via mutation, affecting the subcellular distribution of *GL3* [51]. In this study, the expression patterns of the three types of genes were similar during the critical stage of grain formation, and the expression levels increased in the purple-grain variety Nierumuzha but remained unchanged or decreased in the white-grain variety Kunlun 10. The Y2H and BiFC results revealed that HvnANT1, HvnANT2, and HvWD40-140 can form protein interactions, suggesting that they can form an MBW complex. The results of Zhou et al. [15] showed that *Ant1* and the co-expression of *Ant1* and *Ant2* can activate the expression of the anthocyanin synthesis related structural gene *DFR*. In this study, it was found that the MBW complex (HvANT1, HvANT2, and HvWD40-140) can activate the expression of *HvDFR*. We speculate that *HvnANT1*, *HvnANT2*, and *HvnWD40-140* play an important regulatory role in anthocyanin synthesis in Qingke purple grains, but the mechanism needs to be studied further.

## Conclusion

In this study, the *WD40* family genes in barley were comprehensively analyzed, and a total of 164 *WD40* genes were obtained. They were divided into 11 phylogenetic groups and 14 subfamilies. The HvWD40 family had a highly similar exon–intron structure and motif within the same subgroup, and the regulatory functions of the different subgroups were specific. Based on collinearity analysis, there were 43 pairs of genes between barley and rice and 56 pairs between barley and maize. Go enrichment analysis of the 164 *HvWD40* genes revealed that they were mainly related to biological processes and molecular functions. KEGG analysis revealed that those concentrated in RNA transport pathways were mainly enriched [42–44]. The *HvANT1*, *HvANT2*, and *HvWD40-140* genes were screened based on the analysis of the barley *HvWD40* family identification and RNA-seq results. The expression patterns of the three types of transcription factors were similar during the critical period of grain color formation. HvnANT1 was located in the nucleus and cell membrane, HvnANT2 was located in the nucleus, and HvWD40-140 was located in the chloroplast. Through yeast two-hybrid and bimolecular fluorescence complementarity analyses, it was proven that HvnANT1 in the MYB family and HvnANT2 in the bHLH family can interact with HvWD40-140 in the WD40 family. It was shown that HvnANT1, HvnANT2, and HvWD40-140 form the MBW complex. Moreover, they can regulate the activation of *HvDFR*. These results provide valuable resource for improving our understanding of the regulation of anthocyanin synthesis by an MBW complex in Qingke.

## Methods

### Test materials

The Qingke varieties Nierumuzha and Kunlun 10 were provided by the Academy of Agriculture and Forestry Sciences, Qinghai University.

### Identification and analysis of barley WD40 family members

The WD40 family HMM files were downloaded from the Pfam database (http://pfam.xfam.org/family/PF00400/hmm). The FASTA and GTF files containing candidate barley sequences for the WD40 domains were downloaded from the Gramene database (http://www.gramene.org/). The domain analysis was performed using the Simple Modular Architecture Research Tool (SMART) (http://smart.embl-heidelberg.de/), while the Basic Local Alignment Search Tool (BLAST) alignment of the barley genome (ftp://ftp.gramene.org/pub/gramene/release-63/fasta/hordeum_vulgare/dna/) was performed on the entire genomes of rice (http://rice.plantbiology.msu.edu/pub/data/Eukaryotic_Projects/o_sativa/annotation_dbs/pseudomolecules/version_7.0/all.dir/all.con) and maize (ftp://ftp.ensemblgenomes.org/pub/plants/release43/fasta/zea_mays/dna/Zea_mays.B73_RefGen_v4.dna.toplevel.fa.gz), followed by screening using an E-value of 1e^− 4^ to remove sequences with severe structural domain deletions and duplicate names. We obtained 164 *HvWD40s* family genes. The physicochemical properties of the 164 HvWD40s proteins were analyzed using Expasy Protparma (http://www.expasy.org/tools/protparam.html). The subcellular localization was predicted using CELLO (http://cello.life.nctu.edu.tw). A phylogenetic tree was constructed using MEGA7 and the neighborhood method (NJ) with 1000 bootstrap replicates, then, the phylogenetic tree was drawn using iTOL (https://itol.embl.de/login.cgi). The covariance analysis was performed using MCscanX software. The gene structure and chromosome physical locations of the 164 *HvWD40s* in the GTF file were extracted. The conserved motifs of the protein gene family were predicted using MEME (https://meme-suite.org/meme/doc/meme.html), with a motif maximum of nine and an optimized motif width of 6–50. The cis-acting elements of the *HvWD40s* were analyzed using PlantCARE (http://bioinformatics.psb.ugent.be/webtools/plantcare/html/). The above data were plotted using Tbtools. The protein interactions were predicted using STRING (https://cn.string-db.org/) and Cytoscape 3.5.1. The 164 *HvWD40*s genes were analyzed using gene ontology (http://wego.genomics.org.cn/cgi-bin/wego/index.pl) and the Kyoto Encyclopedia of Genes and Genomes (www.kegg.jp/kegg/kegg1.html) [42–44].

### Candidate gene screening and expression analysis

According to the classification of the grain growth stages provided by Zadoks [52], Nierumuzha and Kunlun 10 grains were collected at different periods of grain color formation: the early milk stage, late milk stage and soft dough stage. RNA-sequencing (RNA-seq) and quantitative real-time fluorescence polymerase chain reaction (qRT-PCR) analysis were performed three times on each sample. The RNA-seq results are available at NCBI (PRJNA815889). Functional annotation of RNA-seq results and identification and analysis of differentially expressed genes with reference to the methodology of Yao et al. [1].

The total RNA was extracted from the grain coat of the Qingke according to the instructions for the plant RNA extraction kit (Takara, Beijing, China). The concentration and purity of the RNA were determined using an ultra-micro nucleic acid protein measuring instrument (NanoPhotometer, Munich, Germany), and the quality was measured using 1.0% agarose gel elect rophoresis. Reverse transcribed complementary DNA (cDNA) was obtained according to the instructions for the cDNA synthesis kit (Takara, Beijing, China) and was stored at − 80℃. Fluorescent quantitative primers were designed according to the National Center for Biotechnology Information (NCBI) (https://www.ncbi.nlm.nih.gov/) (Additional file 5: Table S5). *TC139057* was used as an internal reference for the qRT-PCR analysis. The PCR reaction system, amplification conditions, and relative gene expression were calculated with reference to the methodology of Yao et al. [53].

### Subcellular localization

Introductory cloning and termination vector cloning were conducted via Gatway technology using invitrogen’s Gateway™ BP Clonase™ and Gatway™ LR Clonase™ II Plus reagents (Invitrogen, Shanghai, China), and the subcellular locational expression vectors HvnANT1-GFP, HvnANT2-GFP, and HvWD40-140-GFP were constructed. The subcellular vectors were transformed into DH5α receptor cells (TransGen, Beijing, China), and three single colonies were collected and sent to Tsingke (Tsingke, Xian, China) for sequencing. The plasmids were extracted from the positive colonies, and HvANT1-GFP, HvANT2-GFP, and HvWD40-140-GFP carrying recombinant plasmids were transferred to agrobacterium strain EHA105 (TransGen, Beijing, China) for agrobacterium-mediated tobacco transformation assay. The green fluorescent protein (GFP) was then detected using a laser confocal microscope (Nikon-A1R, Shanghai, China).

### HvANT1, HvANT2, and HvWD40-140 protein interaction assay

By designing specific primers for HvnANT1, HvnANT2, and HvWD40-140, the two null and target fragments of pGAD-T7 and pGBD-T7 were cleaved using the restriction endonucleases EcoR I and BamH I. The target genes were inserted into the pGAD-T7 and pGBD-T7 vectors using T4 ligation and were transformed into DH5α receptor cells (TransGen, Beijing, China). Three single colonies were picked and sent to Tsingke (Tsingke, Xian, China). Successful sequencing revealed that the HvANT1-AD, HvANT1-BD, HvANT2-BD, and HvWD40-140-AD vectors were successfully constructed. The plasmids were extracted from the positive colonies; the HvANT1-AD, HvANT1-BD, HvANT2-BD, and HvWD40-140 carrying the recombinant plasmids were transformed into yeast strain AH109 receptor cells (TransGen, Beijing, China); and HvANT1, HvANT2, and HvWD40-140 were subjected to self-activation verification and protein interaction verification. HvANT1, HvANT2, and HvWD40-140 were validated for self-activation and protein interactions.

The bimolecular fluorescence complementation (BiFC) vectors of HvANT1, HvANT2, and HvWD40-140 were constructed using the yeast two-hybrid (Y2H) method. The target genes were constructed on two vectors, vectors P2YN and P2YC, via double digestion and T4 linkage of restriction enzymes Spe I and Pac I, respectively, and the HvANT1-YFP, HvANT2-YFP, and HvWD40-140-YFP vectors were obtained. The recombinant plasmid was transformed into tobacco leaf cells using the same method described in Sect. 1.4 and were incubated in the dark at 25 °C for 48 h. The yellow fluorescent protein (YFP) was observed under a laser confocal microscope.

### Transcriptional activation of structural genes regulated by the MBW complex

The 2-kb genomic sequence upstream of the *HvDFR* translation start codon was amplified via PCR from genomic DNA and was cloned into pGreenII0800-LUC to generate luciferase reporter constructs. The genomic sequences of *HvANT1*, *HvANT2*, and *HvWD40-140* were cloned into the pGreen 62SK vector under the control of the 35 S promoter and were used as effectors. The constructed vector plasmid was transferred to agrobacterium tumefacien (GV3101) via electro conversion and was cultured at 30℃ for 2 days. The suspension was injected into the lower epidermis of tobacco leaves and cultured under low light for 2 days using the Duo-Lite Luciferase Assay System (Vazyme, Nanjing, China), and firefly luciferase and renilla luciferase tests were performed [54, 55].

## Electronic supplementary material

Below is the link to the electronic supplementary material.

Supplementary Material 1

## Data Availability

WD40 family HMM files from the Pfam database (http://pfam.xfam.org/family/PF00400/hmm). FASTA and GTF files from the Ensembl Genomes (http://www.gramene.org/). Rice genome from Rice Genome Annotation Project (http://rice.plantbiology.msu.edu/pub/data/Eukaryotic_Projects/o_sativa/annotation_dbs/pseudomolecules/version_7.0/all.dir/all.con) and maize genome from Ensembl Genomes (http://www.gramene.org/), The public RNA-seq data from National Center for Biotechnology Information (NCBI). The datasets generated for this study can be found in the Sequence Read Archive (SRA) accession number: SRR18355550, SRR18355551, SRR18355552, SRR18355553, SRR18355554, SRR18355555, SRR18355556, SRR18355557, SRR18355558, SRR18355559, SRR18355560, SRR18355561, SRR18355562, SRR18355563, SRR18355564, SRR18355565, SRR18355566, SRR18355567.

## Abbreviations

WD40: WD repeat
MYB: MYB transcription factors
bHLH: Basic Helix-Loop-Helix
MBW: MYB-bHLH-WD40
GO: Gene Ontology
KEGG: Kyoto Encyclopedia of Genes and Genomes
RNA-seq: Transcriptome sequencing
qRT-PCR: real-time fluorescence PCR
LBGs: Late biosynthetic genes
TT8: Transparent testa 8
TTG1: Transparent testa glabra 1
Y2H: Yeast two-hybrid
BiFC: Bimolecular fluorescent complimentary
MWs: Molecular weights
pIs: Isoelectric point
GRAVY: Total average protein hydrophilicity
HMM: The hidden Markov model
GTF: Gene transfer format
SMART: Simple Modular Architecture Research Tool
N-J: Neighbor-Joining
MCScanX: Multiple Collinearity Scan toolkit
MEME: Multiple Em for Motif Elicitation
NCBI: National Center for Biotechnology Information
GFP: Green fluorescent protein
YFP: Yellow fluorescent protein
DFR: Dihydroflavonol 4-reductase
3-AT: 3-amino-1,2, 4-triazole.
