# Supplementary material for: Genome-wide identification of WD40 transcription factors and their regulation of the MYB-bHLH-WD40 (MBW) complex related to anthocyanin synthesis in Qingke (Hordeum vulgare L. var. nudum Hook. f.)
Source: BMC Genomics. 2023 Apr 4;24:166. doi: 10.1186/s12864-023-09240-5 (PMC10074677; doi:10.1186/s12864-023-09240-5)
Supplement: Supplementary file 1 — Supplementary Material 1 [file 12864_2023_9240_MOESM1_ESM.docx]

| **Additional file 1: Table S1. Distribution of chromosomes of 164 *HvWD40*s** | | | | | |
| --- | --- | --- | --- | --- | --- |
| **No.** | **Gene ID** | **Gene name** | **Chr. No.** | **Start position (bp)** | **End Position (bp)** |
|  |  |  |  |  |  |
| 1 | HORVU1Hr1G004960 | *HvWD40-1* | chr1H | 10739577 | 10746629 |
| 2 | HORVU1Hr1G007930 | *HvWD40-2* | chr1H | 17203138 | 17208768 |
| 3 | HORVU1Hr1G018790 | *HvWD40-3* | chr1H | 70062067 | 70069419 |
| 4 | HORVU1Hr1G020470 | *HvWD40-4* | chr1H | 80827355 | 80832088 |
| 5 | HORVU1Hr1G020620 | *HvWD40-5* | chr1H | 81825284 | 81832874 |
| 6 | HORVU1Hr1G038880 | *HvWD40-6* | chr1H | 271131140 | 271137217 |
| 7 | HORVU1Hr1G039050 | *HvWD40-7* | chr1H | 272999682 | 273010309 |
| 8 | HORVU1Hr1G040250 | *HvWD40-8* | chr1H | 284571341 | 284583896 |
| 9 | HORVU1Hr1G052560 | *HvWD40-9* | chr1H | 389343177 | 389346297 |
| 10 | HORVU1Hr1G055570 | *HvWD40-10* | chr1H | 406989991 | 406995276 |
| 11 | HORVU1Hr1G058470 | *HvWD40-11* | chr1H | 427091045 | 427094211 |
| 12 | HORVU1Hr1G058790 | *HvWD40-12* | chr1H | 428220464 | 428226049 |
| 13 | HORVU1Hr1G060850 | *HvWD40-13* | chr1H | 441911313 | 441920827 |
| 14 | HORVU1Hr1G063720 | *HvWD40-14* | chr1H | 456727346 | 456732891 |
| 15 | HORVU1Hr1G079080 | *HvWD40-15* | chr1H | 522360824 | 522369803 |
| 16 | HORVU1Hr1G081320 | *HvWD40-16* | chr1H | 527455211 | 527459883 |
| 17 | HORVU1Hr1G083710 | *HvWD40-17* | chr1H | 532325138 | 532327766 |
| 18 | HORVU1Hr1G086380 | *HvWD40-18* | chr1H | 537355774 | 537360717 |
| 19 | HORVU1Hr1G090080 | *HvWD40-19* | chr1H | 545249187 | 545257929 |
| 20 | HORVU1Hr1G092950 | *HvWD40-20* | chr1H | 551836459 | 551841100 |
| 21 | HORVU1Hr1G095080 | *HvWD40-21* | chr1H | 557108008 | 557115788 |
| 22 | HORVU2Hr1G011560 | *HvWD40-22* | chr2H | 23187650 | 23191402 |
| 23 | HORVU2Hr1G022250 | *HvWD40-23* | chr2H | 64369423 | 64374434 |
| 24 | HORVU2Hr1G032990 | *HvWD40-24* | chr2H | 130416743 | 130427802 |
| 25 | HORVU2Hr1G037490 | *HvWD40-25* | chr2H | 172357107 | 172358896 |
| 26 | HORVU2Hr1G041020 | *HvWD40-26* | chr2H | 200492263 | 200495577 |
| 27 | HORVU2Hr1G048110 | *HvWD40-27* | chr2H | 267303458 | 267308643 |
| 28 | HORVU2Hr1G053480 | *HvWD40-28* | chr2H | 327883532 | 327908547 |
| 29 | HORVU2Hr1G063680 | *HvWD40-29* | chr2H | 431033218 | 431042858 |
| 30 | HORVU2Hr1G073920 | *HvWD40-30* | chr2H | 534370149 | 534379481 |
| 31 | HORVU2Hr1G083090 | *HvWD40-31* | chr2H | 604171477 | 604173633 |
| 32 | HORVU2Hr1G091010 | *HvWD40-32* | chr2H | 646872648 | 646879635 |
| 33 | HORVU2Hr1G092350 | *HvWD40-33* | chr2H | 652463088 | 652464999 |
| 34 | HORVU2Hr1G093010 | *HvWD40-34* | chr2H | 656338798 | 656339091 |
| 35 | HORVU2Hr1G097080 | *HvWD40-35* | chr2H | 677466856 | 677483600 |
| 36 | HORVU2Hr1G097400 | *HvWD40-36* | chr2H | 678761743 | 678765416 |
| 37 | HORVU2Hr1G103700 | *HvWD40-37* | chr2H | 700723500 | 700726630 |
| 38 | HORVU2Hr1G109500 | *HvWD40-38* | chr2H | 716818007 | 716820421 |
| 39 | HORVU2Hr1G124260 | *HvWD40-39* | chr2H | 758305836 | 758313038 |
| 40 | HORVU3Hr1G003330 | *HvWD40-40* | chr3H | 8299858 | 8337526 |
| 41 | HORVU3Hr1G004510 | *HvWD40-41* | chr3H | 11698452 | 11703468 |
| 42 | HORVU3Hr1G013230 | *HvWD40-42* | chr3H | 28587404 | 28591347 |
| 43 | HORVU3Hr1G017150 | *HvWD40-43* | chr3H | 43245577 | 43251017 |
| 44 | HORVU3Hr1G019880 | *HvWD40-44* | chr3H | 58672552 | 58675948 |
| 45 | HORVU3Hr1G020220 | *HvWD40-45* | chr3H | 62191747 | 62198925 |
| 46 | HORVU3Hr1G022900 | *HvWD40-46* | chr3H | 84066197 | 84081183 |
| 47 | HORVU3Hr1G026100 | *HvWD40-47* | chr3H | 104357762 | 104362030 |
| 48 | HORVU3Hr1G027730 | *HvWD40-48* | chr3H | 120303710 | 120307660 |
| 49 | HORVU3Hr1G031720 | *HvWD40-49* | chr3H | 158834877 | 158836698 |
| 50 | HORVU3Hr1G035450 | *HvWD40-50* | chr3H | 197506208 | 197517206 |
| 51 | HORVU3Hr1G052380 | *HvWD40-51* | chr3H | 380458235 | 380464740 |
| 52 | HORVU3Hr1G052520 | *HvWD40-52* | chr3H | 381485236 | 381496734 |
| 53 | HORVU3Hr1G053250 | *HvWD40-53* | chr3H | 391428086 | 391450502 |
| 54 | HORVU3Hr1G055410 | *HvWD40-54* | chr3H | 411027586 | 411031856 |
| 55 | HORVU3Hr1G063670 | *HvWD40-55* | chr3H | 486900925 | 486903319 |
| 56 | HORVU3Hr1G066930 | *HvWD40-56* | chr3H | 508405447 | 508413405 |
| 57 | HORVU3Hr1G068840 | *HvWD40-57* | chr3H | 521561134 | 521567700 |
| 58 | HORVU3Hr1G076030 | *HvWD40-58* | chr3H | 567513840 | 567518550 |
| 59 | HORVU3Hr1G084470 | *HvWD40-59* | chr3H | 607939786 | 607940801 |
| 60 | HORVU3Hr1G095360 | *HvWD40-60* | chr3H | 649803325 | 649810083 |
| 61 | HORVU3Hr1G098020 | *HvWD40-61* | chr3H | 657450243 | 657456058 |
| 62 | HORVU3Hr1G106030 | *HvWD40-62* | chr3H | 670355396 | 670357153 |
| 63 | HORVU3Hr1G113740 | *HvWD40-63* | chr3H | 688984300 | 688999498 |
| 64 | HORVU3Hr1G115170 | *HvWD40-64* | chr3H | 692797735 | 692801414 |
| 65 | HORVU4Hr1G001430 | *HvWD40-65* | chr4H | 2254894 | 2264900 |
| 66 | HORVU4Hr1G003110 | *HvWD40-66* | chr4H | 6227028 | 6233027 |
| 67 | HORVU4Hr1G003940 | *HvWD40-67* | chr4H | 8815385 | 8824248 |
| 68 | HORVU4Hr1G005810 | *HvWD40-68* | chr4H | 13745734 | 13749602 |
| 69 | HORVU4Hr1G007520 | *HvWD40-69* | chr4H | 19711862 | 19716789 |
| 70 | HORVU4Hr1G009500 | *HvWD40-70* | chr4H | 27033568 | 27039820 |
| 71 | HORVU4Hr1G010910 | *HvWD40-71* | chr4H | 33034985 | 33040285 |
| 72 | HORVU4Hr1G011440 | *HvWD40-72* | chr4H | 35483641 | 35554878 |
| 73 | HORVU4Hr1G011790 | *HvWD40-73* | chr4H | 37206309 | 37212524 |
| 74 | HORVU4Hr1G012010 | *HvWD40-74* | chr4H | 38407615 | 38481098 |
| 75 | HORVU4Hr1G012500 | *HvWD40-75* | chr4H | 41622295 | 41627757 |
| 76 | HORVU4Hr1G017640 | *HvWD40-76* | chr4H | 78942940 | 78947060 |
| 77 | HORVU4Hr1G019090 | *HvWD40-77* | chr4H | 89399904 | 89409349 |
| 78 | HORVU4Hr1G033170 | *HvWD40-78* | chr4H | 229551147 | 229553839 |
| 79 | HORVU4Hr1G034750 | *HvWD40-79* | chr4H | 252632653 | 252637368 |
| 80 | HORVU4Hr1G039720 | *HvWD40-80* | chr4H | 304694447 | 304706541 |
| 81 | HORVU4Hr1G040280 | *HvWD40-81* | chr4H | 309109157 | 309113599 |
| 82 | HORVU4Hr1G046270 | *HvWD40-82* | chr4H | 368050990 | 368052972 |
| 83 | HORVU4Hr1G054000 | *HvWD40-83* | chr4H | 449896709 | 449911537 |
| 84 | HORVU4Hr1G057080 | *HvWD40-84* | chr4H | 479130443 | 479133788 |
| 85 | HORVU4Hr1G067810 | *HvWD40-85* | chr4H | 563078669 | 563087942 |
| 86 | HORVU4Hr1G069350 | *HvWD40-86* | chr4H | 570198175 | 570203753 |
| 87 | HORVU4Hr1G078050 | *HvWD40-87* | chr4H | 606225436 | 606234907 |
| 88 | HORVU4Hr1G079180 | *HvWD40-88* | chr4H | 611470881 | 611475710 |
| 89 | HORVU4Hr1G083410 | *HvWD40-89* | chr4H | 625310642 | 625317299 |
| 90 | HORVU4Hr1G084710 | *HvWD40-90* | chr4H | 629589221 | 629593649 |
| 91 | HORVU4Hr1G086540 | *HvWD40-91* | chr4H | 634606406 | 634610947 |
| 92 | HORVU4Hr1G088390 | *HvWD40-92* | chr4H | 640491514 | 640497028 |
| 93 | HORVU4Hr1G089350 | *HvWD40-93* | chr4H | 642210339 | 642217897 |
| 94 | HORVU5Hr1G002030 | *HvWD40-94* | chr5H | 5685205 | 5702441 |
| 95 | HORVU5Hr1G006390 | *HvWD40-95* | chr5H | 11135617 | 11145146 |
| 96 | HORVU5Hr1G008820 | *HvWD40-96* | chr5H | 19883483 | 19885802 |
| 97 | HORVU5Hr1G010440 | *HvWD40-97* | chr5H | 26002421 | 26243339 |
| 98 | HORVU5Hr1G011850 | *HvWD40-98* | chr5H | 32160557 | 32167234 |
| 99 | HORVU5Hr1G017260 | *HvWD40-99* | chr5H | 66534680 | 66537115 |
| 100 | HORVU5Hr1G019220 | *HvWD40-100* | chr5H | 83507486 | 83510007 |
| 101 | HORVU5Hr1G036690 | *HvWD40-101* | chr5H | 261517327 | 261528031 |
| 102 | HORVU5Hr1G039370 | *HvWD40-102* | chr5H | 286057857 | 286067739 |
| 103 | HORVU5Hr1G041780 | *HvWD40-103* | chr5H | 317166414 | 317170493 |
| 104 | HORVU5Hr1G045070 | *HvWD40-104* | chr5H | 348787158 | 348795816 |
| 105 | HORVU5Hr1G052660 | *HvWD40-105* | chr5H | 412697955 | 412708580 |
| 106 | HORVU5Hr1G057500 | *HvWD40-106* | chr5H | 450800232 | 450808818 |
| 107 | HORVU5Hr1G058430 | *HvWD40-107* | chr5H | 456872503 | 456878265 |
| 108 | HORVU5Hr1G058460 | *HvWD40-108* | chr5H | 456895535 | 456898237 |
| 109 | HORVU5Hr1G060480 | *HvWD40-109* | chr5H | 473976484 | 473982711 |
| 110 | HORVU5Hr1G075320 | *HvWD40-110* | chr5H | 546661026 | 546662677 |
| 111 | HORVU5Hr1G077690 | *HvWD40-111* | chr5H | 553578811 | 553587345 |
| 112 | HORVU5Hr1G088940 | *HvWD40-112* | chr5H | 582938915 | 582945845 |
| 113 | HORVU5Hr1G093830 | *HvWD40-113* | chr5H | 593130812 | 593134115 |
| 114 | HORVU5Hr1G094420 | *HvWD40-114* | chr5H | 595520708 | 595539223 |
| 115 | HORVU5Hr1G094580 | *HvWD40-115* | chr5H | 596263292 | 596267600 |
| 116 | HORVU5Hr1G095960 | *HvWD40-116* | chr5H | 600088785 | 600093342 |
| 117 | HORVU5Hr1G106990 | *HvWD40-117* | chr5H | 625685010 | 625688302 |
| 118 | HORVU5Hr1G114130 | *HvWD40-118* | chr5H | 643180484 | 643218828 |
| 119 | HORVU5Hr1G123080 | *HvWD40-119* | chr5H | 663355222 | 663363898 |
| 120 | HORVU5Hr1G125650 | *HvWD40-120* | chr5H | 669574507 | 669581733 |
| 121 | HORVU6Hr1G039910 | *HvWD40-121* | chr6H | 210470020 | 210497833 |
| 122 | HORVU6Hr1G044360 | *HvWD40-122* | chr6H | 254320621 | 254325910 |
| 123 | HORVU6Hr1G060150 | *HvWD40-123* | chr6H | 399377886 | 399383371 |
| 124 | HORVU6Hr1G063000 | *HvWD40-124* | chr6H | 423364765 | 423440730 |
| 125 | HORVU6Hr1G063700 | *HvWD40-125* | chr6H | 430868992 | 430895765 |
| 126 | HORVU6Hr1G068080 | *HvWD40-126* | chr6H | 471976031 | 471978929 |
| 127 | HORVU6Hr1G071910 | *HvWD40-127* | chr6H | 499742287 | 499751641 |
| 128 | HORVU6Hr1G085720 | *HvWD40-128* | chr6H | 559837986 | 559847272 |
| 129 | HORVU6Hr1G089700 | *HvWD40-129* | chr6H | 570404914 | 570414327 |
| 130 | HORVU7Hr1G000820 | *HvWD40-130* | chr7H | 1482412 | 1486712 |
| 131 | HORVU7Hr1G006140 | *HvWD40-131* | chr7H | 7943128 | 7956995 |
| 132 | HORVU7Hr1G007950 | *HvWD40-132* | chr7H | 10427094 | 10430171 |
| 133 | HORVU7Hr1G016770 | *HvWD40-133* | chr7H | 21458034 | 21464788 |
| 134 | HORVU7Hr1G019930 | *HvWD40-134* | chr7H | 27119476 | 27129693 |
| 135 | HORVU7Hr1G027670 | *HvWD40-135* | chr7H | 49445156 | 49454463 |
| 136 | HORVU7Hr1G039810 | *HvWD40-136* | chr7H | 106246048 | 106248445 |
| 137 | HORVU7Hr1G045240 | *HvWD40-137* | chr7H | 142388902 | 142396963 |
| 138 | HORVU7Hr1G045450 | *HvWD40-138* | chr7H | 144178408 | 144184989 |
| 139 | HORVU7Hr1G048900 | *HvWD40-139* | chr7H | 168923562 | 168926887 |
| 140 | HORVU7Hr1G058730 | *HvWD40-140* | chr7H | 260598567 | 260607607 |
| 141 | HORVU7Hr1G065740 | *HvWD40-141* | chr7H | 329597135 | 329612930 |
| 142 | HORVU7Hr1G066930 | *HvWD40-142* | chr7H | 346181113 | 346195444 |
| 143 | HORVU7Hr1G073410 | *HvWD40-143* | chr7H | 415781576 | 415787023 |
| 144 | HORVU7Hr1G074240 | *HvWD40-144* | chr7H | 423386388 | 423392615 |
| 145 | HORVU7Hr1G077320 | *HvWD40-145* | chr7H | 453525331 | 453531726 |
| 146 | HORVU7Hr1G080210 | *HvWD40-146* | chr7H | 474089873 | 474106267 |
| 147 | HORVU7Hr1G084630 | *HvWD40-147* | chr7H | 509439437 | 509447258 |
| 148 | HORVU7Hr1G085500 | *HvWD40-148* | chr7H | 517019396 | 517024657 |
| 149 | HORVU7Hr1G087940 | *HvWD40-149* | chr7H | 530973500 | 530982179 |
| 150 | HORVU7Hr1G101230 | *HvWD40-150* | chr7H | 607940680 | 607950134 |
| 151 | HORVU7Hr1G105700 | *HvWD40-151* | chr7H | 617499832 | 617515666 |
| 152 | HORVU7Hr1G111780 | *HvWD40-152* | chr7H | 634618189 | 634622621 |
| 153 | HORVU7Hr1G113760 | *HvWD40-153* | chr7H | 638085727 | 638105117 |
| 154 | HORVU7Hr1G115460 | *HvWD40-154* | chr7H | 641612789 | 641621530 |
| 155 | HORVU7Hr1G117550 | *HvWD40-155* | chr7H | 645364846 | 645367176 |
| 156 | HORVU7Hr1G118690 | *HvWD40-156* | chr7H | 648196415 | 648199087 |
| 157 | HORVU0Hr1G000250 | *HvWD40-157* | chrUn | 697306 | 700408 |
| 158 | HORVU0Hr1G006640 | *HvWD40-158* | chrUn | 38453862 | 38461779 |
| 159 | HORVU0Hr1G008690 | *HvWD40-159* | chrUn | 52121769 | 52131080 |
| 160 | HORVU0Hr1G016450 | *HvWD40-160* | chrUn | 88366914 | 88373289 |
| 161 | HORVU0Hr1G027010 | *HvWD40-161* | chrUn | 143301203 | 143305417 |
| 162 | HORVU0Hr1G027960 | *HvWD40-162* | chrUn | 148493160 | 148494756 |
| 163 | HORVU0Hr1G028010 | *HvWD40-163* | chrUn | 148632088 | 148639030 |
| 164 | HORVU0Hr1G030920 | *HvWD40-164* | chrUn | 181233734 | 181241076 |

| **Additional file 2: Table S2. Characteristic features of 164 HvWD40s** | | | | | | | |  |
| --- | --- | --- | --- | --- | --- | --- | --- | --- |
| Gene ID | Gene | pI | Protein length | Mw | Index of instability | Hydrophilic index | Fat index | Predicted Subcellular location |
| HORVU1Hr1G004960 | HvWD40-1 | 9.25 | 409 | 45328.05 | 48.19 | -0.481 | 68.00 | Chloroplast |
| HORVU1Hr1G007930 | HvWD40-2 | 6.42 | 263 | 29944.26 | 35.55 | -0.127 | 83.76 | Nucleus |
| HORVU1Hr1G018790 | HvWD40-3 | 8.39 | 412 | 46470.19 | 47.89 | 0.474 | 71.43 | Nucleus |
| HORVU1Hr1G020470 | HvWD40-4 | 8.51 | 229 | 24854.43 | 52.66 | -0.245 | 78.43 | Chloroplast |
| HORVU1Hr1G020620 | HvWD40-5 | 6.39 | 836 | 91410.34 | 41.54 | -0.079 | 85.01 | Nucleus |
| HORVU1Hr1G038880 | HvWD40-6 | 5.2 | 261 | 28954.86 | 50.45 | -0.067 | 97.2 | Cytoplasm |
| HORVU1Hr1G039050 | HvWD40-7 | 5.87 | 527 | 57684.42 | 29.68 | -0.278 | 84.02 | Cytoplasm |
| HORVU1Hr1G040250 | HvWD40-8 | 6.63 | 481 | 54005.40 | 39.12 | -0.169 | 86.76 | Nucleus |
| HORVU1Hr1G052560 | HvWD40-9 | 6.08 | 523 | 57701.58 | 44.21 | -0.499 | 73.50 | Chloroplast |
| HORVU1Hr1G055570 | HvWD40-10 | 4.96 | 467 | 52423.70 | 42.98 | -0.134 | 81.86 | Cytoplasm |
| HORVU1Hr1G058470 | HvWD40-11 | 8.62 | 226 | 25020.34 | 64.22 | -0.433 | 68.19 | Chloroplast |
| HORVU1Hr1G058790 | HvWD40-12 | 8.47 | 876 | 98065.71 | 48.00 | -0.122 | 97.41 | Nucleus |
| HORVU1Hr1G060850 | HvWD40-13 | 5.92 | 250 | 27908.85 | 38.99 | -0.441 | 76.72 | Cytoplasm |
| HORVU1Hr1G063720 | HvWD40-14 | 4.87 | 611 | 66948.09 | 34.9 | -0.042 | 96.37 | Chloroplast |
| HORVU1Hr1G079080 | HvWD40-15 | 8.89 | 390 | 42848.10 | 56.54 | -0.369 | 75.85 | Nucleus |
| HORVU1Hr1G081320 | HvWD40-16 | 4.7 | 399 | 43431.55 | 34.60 | -0.308 | 84.11 | Chloroplast |
| HORVU1Hr1G083710 | HvWD40-17 | 8.71 | 351 | 38452.64 | 49.22 | -0.199 | 88.80 | Nucleus |
| HORVU1Hr1G086380 | HvWD40-18 | 6.35 | 883 | 97133.38 | 42.82 | -0.100 | 85.87 | Chloroplast |
| HORVU1Hr1G090080 | HvWD40-19 | 6.34 | 970 | 108662.8 | 49.56 | -0.587 | 72.57 | Nucleus |
| HORVU1Hr1G092950 | HvWD40-20 | 4.66 | 558 | 60346.10 | 43.75 | -0.482 | 70.11 | Chloroplast |
| HORVU1Hr1G095080 | HvWD40-21 | 8.62 | 503 | 55225.8 | 33.8 | -0.304 | 80.46 | Chloroplast |
| HORVU2Hr1G011560 | HvWD40-22 | 8 | 347 | 38850.99 | 32.04 | -0.194 | 80.00 | Nucleus |
| HORVU2Hr1G022250 | HvWD40-23 | 4.49 | 400 | 43204.17 | 46.85 | -0.394 | 69.55 | Nucleus |
| HORVU2Hr1G032990 | HvWD40-24 | 6.93 | 289 | 32038.04 | 34.49 | -0.268 | 79.34 | Nucleus |
| HORVU2Hr1G037490 | HvWD40-25 | 8.82 | 207 | 22686.95 | 22.25 | 0.133 | 93.67 | Chloroplast |
| HORVU2Hr1G041020 | HvWD40-26 | 8.73 | 262 | 28169.05 | 40.36 | -0.065 | 83.40 | Chloroplast |
| HORVU2Hr1G048110 | HvWD40-27 | 8.98 | 167 | 18392.02 | 29.70 | -0.149 | 78.80 | Cytoskeleton |
| HORVU2Hr1G053480 | HvWD40-28 | 5.66 | 557 | 62514.21 | 32.10 | -0.582 | 72.62 | Nucleus |
| HORVU2Hr1G063680 | HvWD40-29 | 6.29 | 292 | 31220.19 | 40.14 | -0.062 | 85.14 | Cytoplasm |
| HORVU2Hr1G073920 | HvWD40-30 | 8.43 | 683 | 75751.73 | 39.73 | -0.463 | 71.89 | Nucleus |
| HORVU2Hr1G083090 | HvWD40-31 | 9.23 | 449 | 46497.11 | 45.45 | -0.062 | 83.32 | Mitochondrial inner membrane |
| HORVU2Hr1G091010 | HvWD40-32 | 8.78 | 620 | 68617.49 | 59.59 | -0.654 | 64.31 | Nucleus |
| HORVU2Hr1G092350 | HvWD40-33 | 5.23 | 129 | 14664.4 | 29.52 | -0.396 | 79.30 | Cytoplasm |
| HORVU2Hr1G093010 | HvWD40-34 | 7.68 | 67 | 7391.41 | 40.61 | -0.210 | 75.67 | Chloroplast |
| HORVU2Hr1G097080 | HvWD40-35 | 6.31 | 1819 | 200586.60 | 50.34 | -0.234 | 86.68 | Nucleus |
| HORVU2Hr1G097400 | HvWD40-36 | 5.89 | 336 | 37320.26 | 37.69 | -0.289 | 80.68 | Nucleus |
| HORVU2Hr1G103700 | HvWD40-37 | 8.11 | 603 | 66600.63 | 45.77 | -0.273 | 78.14 | Chloroplast |
| HORVU2Hr1G109500 | HvWD40-38 | 5.63 | 317 | 33964.42 | 44.80 | -0.082 | 81.86 | Cytoplasm |
| HORVU2Hr1G124260 | HvWD40-39 | 9.77 | 578 | 65002.22 | 39.26 | -0.632 | 74.58 | Chloroplast |
| HORVU3Hr1G003330 | HvWD40-40 | 5.95 | 203 | 23140.43 | 34.48 | -0.037 | 95.96 | Cytoplasm |
| HORVU3Hr1G004510 | HvWD40-41 | 6.21 | 611 | 66100.27 | 31.06 | -0.234 | 78.51 | Cytoplasm |
| HORVU3Hr1G013230 | HvWD40-42 | 4.32 | 398 | 42914.2 | 38.02 | -0.138 | 85.00 | Chloroplast |
| HORVU3Hr1G017150 | HvWD40-43 | 8.01 | 417 | 46396.03 | 40.70 | -0.20 | 78.30 | Nucleus |
| HORVU3Hr1G019880 | HvWD40-44 | 7.13 | 259 | 28126.57 | 38.20 | 0.011 | 91.12 | Cytoplasm |
| HORVU3Hr1G020220 | HvWD40-45 | 8.99 | 482 | 53868.18 | 39.59 | -0.234 | 87.18 | Nucleus |
| HORVU3Hr1G022900 | HvWD40-46 | 6.4 | 977 | 106969.2 | 58.36 | -0.551 | 69.41 | Chloroplast |
| HORVU3Hr1G026100 | HvWD40-47 | 6.88 | 269 | 28720.30 | 28.56 | -0.152 | 76.06 | Chloroplast |
| HORVU3Hr1G027730 | HvWD40-48 | 5.78 | 305 | 33274.34 | 33.14 | -0.294 | 75.11 | Cytoplasm |
| HORVU3Hr1G031720 | HvWD40-49 | 7.1 | 507 | 53679.67 | 52.56 | -0.172 | 77.32 | Cytoplasm |
| HORVU3Hr1G035450 | HvWD40-50 | 9.04 | 479 | 52531.85 | 38.10 | -0.364 | 76.83 | Nucleus |
| HORVU3Hr1G052380 | HvWD40-51 | 5.92 | 504 | 56017.76 | 50.56 | -0.457 | 74.56 | Nucleus |
| HORVU3Hr1G052520 | HvWD40-52 | 6.13 | 1385 | 149093.10 | 46.02 | -0.105 | 92.32 | Plasmalemma |
| HORVU3Hr1G053250 | HvWD40-53 | 6.25 | 910 | 100006.00 | 61.16 | -0.697 | 67.34 | Nucleus |
| HORVU3Hr1G055410 | HvWD40-54 | 4.9 | 323 | 35259.78 | 48.03 | -0.140 | 84.55 | Chloroplast |
| HORVU3Hr1G063670 | HvWD40-55 | 5.96 | 307 | 33543.67 | 38.93 | -0.099 | 84.72 | Cytoplasm |
| HORVU3Hr1G066930 | HvWD40-56 | 6.17 | 509 | 56081.32 | 46.86 | -0.572 | 67.47 | Chloroplast |
| HORVU3Hr1G068840 | HvWD40-57 | 9.59 | 277 | 31267.68 | 40.41 | -0.229 | 77.69 | Nucleus |
| HORVU3Hr1G076030 | HvWD40-58 | 9.83 | 354 | 38817.02 | 56.28 | -0.38 | 73.59 | Chloroplast |
| HORVU3Hr1G084470 | HvWD40-59 | 9.17 | 196 | 22109.95 | 63.10 | -0.576 | 65.15 | Nucleus |
| HORVU3Hr1G095360 | HvWD40-60 | 4.56 | 505 | 54721.55 | 40.54 | -0.415 | 74.95 | Chloroplast |
| HORVU3Hr1G098020 | HvWD40-61 | 5.52 | 508 | 54948.93 | 59.28 | -0.413 | 74.15 | Nucleus |
| HORVU3Hr1G106030 | HvWD40-62 | 9.34 | 436 | 45834.08 | 45.21 | -0.070 | 79.98 | Cytoplasm |
| HORVU3Hr1G113740 | HvWD40-63 | 5.62 | 768 | 86862.02 | 42.68 | -0.180 | 91.71 | Nucleus |
| HORVU3Hr1G115170 | HvWD40-64 | 10.29 | 587 | 63322.30 | 57.29 | -0.533 | 67.21 | Chloroplast |
| HORVU4Hr1G001430 | HvWD40-65 | 6.88 | 995 | 109039.10 | 38.89 | -0.387 | 73.83 | Cytoplasm |
| HORVU4Hr1G003110 | HvWD40-66 | 6.7 | 443 | 47697.41 | 32.49 | -0.255 | 77.49 | Chloroplast |
| HORVU4Hr1G003940 | HvWD40-67 | 5.94 | 189 | 20958.77 | 26.45 | -0.119 | 82.01 | Cytoplasm |
| HORVU4Hr1G005810 | HvWD40-68 | 8.62 | 306 | 33029.24 | 40.89 | -0.281 | 81.54 | Chloroplast |
| HORVU4Hr1G007520 | HvWD40-69 | 6.61 | 1122 | 125236.00 | 33.87 | -0.237 | 90.21 | Mitochondria |
| HORVU4Hr1G009500 | HvWD40-70 | 7.77 | 347 | 37367.12 | 37.00 | -0.230 | 79.05 | Chloroplast |
| HORVU4Hr1G010910 | HvWD40-71 | 9.08 | 893 | 98110.02 | 46.77 | -0.382 | 74.59 | Nucleus |
| HORVU4Hr1G011440 | HvWD40-72 | 4.4 | 296 | 31546.49 | 46.18 | -0.293 | 60.61 | Nucleus |
| HORVU4Hr1G011790 | HvWD40-73 | 9.59 | 531 | 59758.11 | 32.85 | -0.598 | 77.70 | Cytoplasm |
| HORVU4Hr1G012010 | HvWD40-74 | 9.39 | 351 | 37490.69 | 27.03 | -0.496 | 68.95 | Cytoplasm |
| HORVU4Hr1G012500 | HvWD40-75 | 9.62 | 432 | 48116.86 | 54.28 | -0.350 | 78.08 | Chloroplast |
| HORVU4Hr1G017640 | HvWD40-76 | 6.8 | 661 | 71870.12 | 55.66 | -0.621 | 66.17 | Chloroplast |
| HORVU4Hr1G019090 | HvWD40-77 | 6.78 | 594 | 63229.78 | 43.70 | -0.274 | 71.77 | Endoplasmic reticulum |
| HORVU4Hr1G033170 | HvWD40-78 | 6.23 | 342 | 36175.91 | 38.23 | -0.049 | 86.75 | Mitochondria |
| HORVU4Hr1G034750 | HvWD40-79 | 5.86 | 504 | 55998.12 | 42.04 | -0.446 | 78.47 | Chloroplast |
| HORVU4Hr1G039720 | HvWD40-80 | 6.72 | 576 | 64579.39 | 52.74 | -0.652 | 65.05 | Nucleus |
| HORVU4Hr1G040280 | HvWD40-81 | 10 | 273 | 30360.57 | 45.56 | -0.434 | 83.66 | Chloroplast |
| HORVU4Hr1G046270 | HvWD40-82 | 7.74 | 139 | 15330.76 | 34.16 | -0.059 | 95.40 | Chloroplast |
| HORVU4Hr1G054000 | HvWD40-83 | 6.5 | 515 | 58021.26 | 58.68 | -0.577 | 71.98 | Nucleus |
| HORVU4Hr1G057080 | HvWD40-84 | 6.4 | 371 | 41776.97 | 48.32 | -0.526 | 70.65 | Nucleus |
| HORVU4Hr1G067810 | HvWD40-85 | 9.12 | 400 | 43874.39 | 46.73 | -0.447 | 64.62 | Chloroplast |
| HORVU4Hr1G069350 | HvWD40-86 | 4.86 | 588 | 64015.06 | 40.16 | -0.346 | 82.30 | Nucleus |
| HORVU4Hr1G078050 | HvWD40-87 | 8.16 | 422 | 46486.72 | 54.79 | -0.444 | 76.92 | Nucleus |
| HORVU4Hr1G079180 | HvWD40-88 | 9.21 | 457 | 48103.86 | 38.08 | -0.216 | 78.12 | Chloroplast |
| HORVU4Hr1G083410 | HvWD40-89 | 6.87 | 1013 | 112046.50 | 43.77 | -0.313 | 86.35 | Cytoplasm |
| HORVU4Hr1G084710 | HvWD40-90 | 9.79 | 563 | 60272.72 | 47.54 | -0.442 | 66.64 | Nucleus |
| HORVU4Hr1G086540 | HvWD40-91 | 5.94 | 757 | 83393.86 | 55.97 | -0.487 | 71.86 | Nucleus |
| HORVU4Hr1G088390 | HvWD40-92 | 6.54 | 1218 | 135810.90 | 33.34 | -0.234 | 88.48 | Mitochondria |
| HORVU4Hr1G089350 | HvWD40-93 | 6.1 | 1033 | 113734.60 | 38.11 | -0.081 | 92.59 | Chloroplast |
| HORVU5Hr1G002030 | HvWD40-94 | 6.75 | 1715 | 191144.30 | 49.69 | -0.669 | 66.85 | Nucleus |
| HORVU5Hr1G006390 | HvWD40-95 | 5.75 | 906 | 99631.72 | 47.11 | -0.102 | 86.60 | Nucleus |
| HORVU5Hr1G008820 | HvWD40-96 | 9.23 | 348 | 36800.95 | 45.08 | -0.036 | 89.02 | Chloroplast |
| HORVU5Hr1G010440 | HvWD40-97 | 6.45 | 303 | 33215.66 | 45.48 | -0.248 | 85.38 | Chloroplast |
| HORVU5Hr1G011850 | HvWD40-98 | 9.02 | 436 | 47439.37 | 33.56 | -0.098 | 84.95 | Chloroplast |
| HORVU5Hr1G017260 | HvWD40-99 | 11.43 | 521 | 56259.55 | 57.08 | -0.505 | 76.83 | Chloroplast |
| HORVU5Hr1G019220 | HvWD40-100 | 6.95 | 149 | 17318.95 | 22.70 | -0.110 | 80.34 | Cytoplasm |
| HORVU5Hr1G036690 | HvWD40-101 | 6.26 | 388 | 42301.60 | 52.46 | -0.224 | 74.59 | Chloroplast |
| HORVU5Hr1G039370 | HvWD40-102 | 6.78 | 561 | 60998.57 | 37.58 | -0.291 | 80.82 | Nucleus |
| HORVU5Hr1G041780 | HvWD40-103 | 8.81 | 476 | 52615.58 | 49.92 | -0.414 | 73.78 | Nucleus |
| HORVU5Hr1G045070 | HvWD40-104 | 5.23 | 334 | 36199.65 | 43.08 | -0.246 | 70.03 | Cytoplasm |
| HORVU5Hr1G052660 | HvWD40-105 | 6.71 | 775 | 82993.72 | 48.55 | -0.394 | 77.05 | Nucleus |
| HORVU5Hr1G057500 | HvWD40-106 | 5.16 | 390 | 41529.65 | 46.07 | -0.049 | 80.15 | Nucleus |
| HORVU5Hr1G058430 | HvWD40-107 | 5.38 | 340 | 37020.42 | 43.65 | -0.371 | 70.85 | Cytoplasm |
| HORVU5Hr1G058460 | HvWD40-108 | 6.35 | 292 | 32258.36 | 32.12 | -0.266 | 71.82 | Chloroplast |
| HORVU5Hr1G060480 | HvWD40-109 | 6.38 | 1010 | 108561.40 | 43.52 | -0.348 | 75.87 | Chloroplast |
| HORVU5Hr1G075320 | HvWD40-110 | 8.69 | 405 | 44927.01 | 38.13 | -0.372 | 83.26 | Cytoplasm |
| HORVU5Hr1G077690 | HvWD40-111 | 9.18 | 753 | 82732.19 | 58.72 | -52.48 | 52.48 | Chloroplast |
| HORVU5Hr1G088940 | HvWD40-112 | 6.12 | 670 | 76785.46 | 47.42 | -0.729 | 74.18 | Chloroplast |
| HORVU5Hr1G093830 | HvWD40-113 | 8.28 | 479 | 51136.12 | 47.91 | -0.346 | 71.46 | Cytoplasm |
| HORVU5Hr1G094420 | HvWD40-114 | 5.56 | 3288 | 366074.50 | 43.15 | -0.159 | 92.95 | Plasmalemma |
| HORVU5Hr1G094580 | HvWD40-115 | 5.49 | 432 | 46376.65 | 33.42 | -0.047 | 92.13 | Chloroplast |
| HORVU5Hr1G095960 | HvWD40-116 | 8.35 | 418 | 45483.32 | 35.21 | -0.259 | 76.56 | Cytoplasm |
| HORVU5Hr1G106990 | HvWD40-117 | 6.62 | 357 | 39317.33 | 43.81 | 0.079 | 91.20 | Chloroplast |
| HORVU5Hr1G114130 | HvWD40-118 | 5.94 | 902 | 101370.8 | 49.40 | -0.254 | 86.54 | Nucleus |
| HORVU5Hr1G123080 | HvWD40-119 | 6.86 | 751 | 81547.70 | 54.67 | -0.276 | 77.87 | Nucleus |
| HORVU5Hr1G125650 | HvWD40-120 | 8.94 | 817 | 89531.96 | 46.56 | -0.697 | 63.94 | Nucleus |
| HORVU6Hr1G039910 | HvWD40-121 | 6.55 | 521 | 58332.12 | 51.72 | -0.582 | 72.46 | Chloroplast |
| HORVU6Hr1G044360 | HvWD40-122 | 4.7 | 415 | 44045.86 | 39.62 | -0.257 | 79.73 | Cytoskeleton |
| HORVU6Hr1G060150 | HvWD40-123 | 6.3 | 467 | 51553.42 | 58.67 | -0.576 | 67.00 | Nucleus |
| HORVU6Hr1G063000 | HvWD40-124 | 7.25 | 318 | 35088.34 | 42.47 | -0.352 | 73.18 | Cytoplasm |
| HORVU6Hr1G063700 | HvWD40-125 | 5.52 | 2862 | 313858.5 | 51.28 | -0.136 | 88.75 | Plasmalemma |
| HORVU6Hr1G068080 | HvWD40-126 | 6.97 | 475 | 52010.32 | 47.14 | -0.422 | 47.14 | Chloroplast |
| HORVU6Hr1G071910 | HvWD40-127 | 5.9 | 1122 | 121517.5 | 48.51 | -0.386 | 79.48 | Chloroplast |
| HORVU6Hr1G085720 | HvWD40-128 | 8.2 | 1521 | 168250.4 | 48.43 | -0.253 | 86.10 | Chloroplast |
| HORVU6Hr1G089700 | HvWD40-129 | 6.44 | 223 | 24823.10 | 38.51 | -0.105 | 81.66 | Chloroplast |
| HORVU7Hr1G000820 | HvWD40-130 | 5.36 | 439 | 47988.39 | 51.71 | -0.385 | 72.00 | Nucleus |
| HORVU7Hr1G006140 | HvWD40-131 | 6.09 | 738 | 84158.3 | 40.09 | -0.332 | 78.04 | Chloroplast |
| HORVU7Hr1G007950 | HvWD40-132 | 6.23 | 264 | 29432.36 | 26.24 | -0.172 | 85.27 | Chloroplast |
| HORVU7Hr1G016770 | HvWD40-133 | 8.09 | 253 | 28269.49 | 38.05 | 0.019 | 96.68 | Chloroplast |
| HORVU7Hr1G019930 | HvWD40-134 | 5.24 | 972 | 109491.10 | 40.41 | -0.330 | 83.67 | Chloroplast |
| HORVU7Hr1G027670 | HvWD40-135 | 5.89 | 493 | 54665.61 | 30.18 | -0.370 | 77.32 | Chloroplast |
| HORVU7Hr1G039810 | HvWD40-136 | 8.4 | 384 | 40760.04 | 50.20 | -0.065 | 83.80 | Nucleus |
| HORVU7Hr1G045240 | HvWD40-137 | 6.01 | 771 | 83489.45 | 42.09 | -0.378 | 79.29 | Mitochondria |
| HORVU7Hr1G045450 | HvWD40-138 | 4.95 | 526 | 58957.34 | 42.52 | -0.525 | 71.22 | Chloroplast |
| HORVU7Hr1G048900 | HvWD40-139 | 5.82 | 345 | 38865.54 | 31.61 | -0.279 | 74.61 | Nucleus |
| HORVU7Hr1G058730 | HvWD40-140 | 6.23 | 648 | 72717.35 | 43.30 | -0.463 | 76.39 | Nucleus |
| HORVU7Hr1G065740 | HvWD40-141 | 4.69 | 444 | 50724.59 | 37.43 | -0.427 | 78.76 | Chloroplast |
| HORVU7Hr1G066930 | HvWD40-142 | 6.53 | 673 | 73499.11 | 43.04 | -0.304 | 77.85 | Chloroplast |
| HORVU7Hr1G073410 | HvWD40-143 | 6.48 | 476 | 52741.83 | 43.77 | -0.321 | 76.47 | Cytoplasm |
| HORVU7Hr1G074240 | HvWD40-144 | 5.98 | 595 | 65947.64 | 47.11 | -0.340 | 86.66 | Nucleus |
| HORVU7Hr1G077320 | HvWD40-145 | 8.57 | 404 | 44691.09 | 45.14 | -0.312 | 78.00 | Chloroplast |
| HORVU7Hr1G080210 | HvWD40-146 | 6.04 | 1643 | 182616.1 | 55.34 | -0.114 | 90.07 | Cytoplasm |
| HORVU7Hr1G084630 | HvWD40-147 | 5.77 | 342 | 37038.42 | 41.23 | -0.343 | 69.04 | Cytoplasm |
| HORVU7Hr1G085500 | HvWD40-148 | 5.77 | 417 | 45606.57 | 48.12 | -0.122 | 93.07 | Cytoplasm |
| HORVU7Hr1G087940 | HvWD40-149 | 5.49 | 526 | 58834.3 | 43.88 | -0.570 | 72.15 | Nucleus |
| HORVU7Hr1G101230 | HvWD40-150 | 4.78 | 338 | 36920.72 | 30.57 | -0.410 | 70.09 | Chloroplast |
| HORVU7Hr1G105700 | HvWD40-151 | 6.4 | 1529 | 172253.90 | 45.67 | -0.247 | 89.90 | Nucleus |
| HORVU7Hr1G111780 | HvWD40-152 | 7.65 | 346 | 38420.55 | 39.65 | -0.464 | 73.58 | Chloroplast |
| HORVU7Hr1G113760 | HvWD40-153 | 6.43 | 656 | 73151.71 | 42.19 | -0.42 | 80.29 | Nucleus |
| HORVU7Hr1G115460 | HvWD40-154 | 9.08 | 539 | 59782.69 | 42.24 | -0.064 | 88.11 | Chloroplast |
| HORVU7Hr1G117550 | HvWD40-155 | 9.34 | 545 | 59372.59 | 41.22 | -0.211 | 83.45 | Chloroplast |
| HORVU7Hr1G118690 | HvWD40-156 | 4.24 | 498 | 55128.97 | 33.56 | -0.643 | 66.69 | Nucleus |
| HORVU0Hr1G000250 | HvWD40-157 | 6.78 | 205 | 23837.82 | 48.46 | -0.556 | 65.12 | Nucleus |
| HORVU0Hr1G006640 | HvWD40-158 | 6.65 | 455 | 51283.63 | 44.20 | -0.284 | 84.44 | Nucleus |
| HORVU0Hr1G008690 | HvWD40-159 | 6.68 | 1136 | 125137.00 | 41.28 | -0.298 | 80.22 | Endoplasmic reticulum |
| HORVU0Hr1G016450 | HvWD40-160 | 5.01 | 377 | 40578.79 | 52.93 | -0.124 | 86.02 | Chloroplast |
| HORVU0Hr1G027010 | HvWD40-161 | 7.84 | 359 | 39475.5 | 46.76 | -0.226 | 79.19 | Vacuole |
| HORVU0Hr1G027960 | HvWD40-162 | 9.92 | 279 | 30122.57 | 58.82 | -0.349 | 74.95 | Chloroplast |
| HORVU0Hr1G028010 | HvWD40-163 | 6.12 | 670 | 76785.46 | 47.42 | -0.729 | 74.18 | Chloroplast |
| HORVU0Hr1G030920 | HvWD40-164 | 5.56 | 737 | 83442.4 | 51.79 | -0.762 | 71.03 | Chloroplast |

| **Additional file 3: Table S3: Orthologous relationships between *HvWD40* genes and *WD40* genes in *Oryza sativa* and *Zea mays*** | | | |
| --- | --- | --- | --- |
| ***Hordeum vulgare***  Chromosome Gene Name | | ***Oryza sativa***  Chromosome Gene Name | |
| Hvchr1H | HORVU1Hr1G052560 | OsChr10 | Os10t0544500 |
| Hvchr1H | HORVU1Hr1G083710 | OsChr1 | Os01t0686800 |
| Hvchr1H | HORVU1Hr1G083710 | OsChr5 | Os05t0552300 |
| Hvchr2H | HORVU2Hr1G091010 | OsChr2 | Os02t0638900 |
| Hvchr2H | HORVU2Hr1G091010 | OsChr4 | Os04t0529400 |
| Hvchr2H | HORVU2Hr1G097080 | OsChr4 | Os04t0555500 |
| Hvchr3H | HORVU3Hr1G004510 | OsChr1 | Os01t0125800 |
| Hvchr3H | HORVU3Hr1G020220 | OsChr1 | Os01t0185400 |
| Hvchr3H | HORVU3Hr1G022900 | OsChr1 | Os01t0177100 |
| Hvchr3H | HORVU3Hr1G031720 | OsChr1 | Os01t0232200 |
| Hvchr3H | HORVU3Hr1G055410 | OsChr1 | Os01t0620100 |
| Hvchr3H | HORVU3Hr1G066930 | OsChr1 | Os01t0710000 |
| Hvchr3H | HORVU3Hr1G098020 | OsChr1 | Os01t0934000 |
| Hvchr3H | HORVU3Hr1G106030 | OsChr1 | Os01t0946100 |
| Hvchr4H | HORVU4Hr1G001430 | OsChr9 | Os09t0567700 |
| Hvchr4H | HORVU4Hr1G009500 | OsChr3 | Os03t0725400 |
| Hvchr4H | HORVU4Hr1G010910 | OsChr3 | Os03t0735100 |
| Hvchr4H | HORVU4Hr1G019090 | OsChr11 | Os11t0176000 |
| Hvchr4H | HORVU4Hr1G019090 | OsChr12 | Os12t0172500 |
| Hvchr4H | HORVU4Hr1G033170 | OsChr11 | Os11t0660300 |
| Hvchr4H | HORVU4Hr1G069350 | OsChr3 | Os03t0207900 |
| Hvchr4H | HORVU4Hr1G079180 | OsChr3 | Os03t0145600 |
| Hvchr4H | HORVU4Hr1G084710 | OsChr3 | Os03t0123300 |
| Hvchr4H | HORVU4Hr1G086540 | OsChr3 | Os03t0115400 |
| Hvchr5H | HORVU5Hr1G008820 | OsChr12 | Os12t0615900 |
| Hvchr5H | HORVU5Hr1G017260 | OsChr12 | Os12t0594000 |
| Hvchr5H | HORVU5Hr1G017260 | OsChr3 | Os03t0625300 |
| Hvchr5H | HORVU5Hr1G093830 | OsChr3 | Os03t0738700 |
| Hvchr5H | HORVU5Hr1G093830 | OsChr3 | Os03t0625300 |
| Hvchr5H | HORVU5Hr1G094580 | OsChr3 | Os03t0746800 |
| Hvchr6H | HORVU6Hr1G085720 | OsChr2 | Os02t0796700 |
| Hvchr7H | HORVU7Hr1G019930 | OsChr6 | Os06t0143900 |
| Hvchr7H | HORVU7Hr1G027670 | OsChr6 | Os06t0171900 |
| Hvchr7H | HORVU7Hr1G039810 | OsChr2 | Os02t0740900 |
| Hvchr7H | HORVU7Hr1G039810 | OsChr6 | Os06t0238700 |
| Hvchr7H | HORVU7Hr1G045450 | OsChr2 | Os02t0721600 |
| Hvchr7H | HORVU7Hr1G073410 | OsChr8 | Os08t0137100 |
| Hvchr7H | HORVU7Hr1G084630 | OsChr6 | Os06t0598900 |
| Hvchr7H | HORVU7Hr1G087940 | OsChr2 | Os02t0224200 |
| Hvchr7H | HORVU7Hr1G087940 | OsChr6 | Os06t0563300 |
| Hvchr7H | HORVU7Hr1G111780 | OsChr6 | Os06t0653800 |
| Hvchr7H | HORVU7Hr1G113760 | OsChr6 | Os06t0649500 |
| HvchrUn | HORVU0Hr1G008690 | OsChr3 | Os03t0254700 |
|  |  |  |  |
| ***Hordeum vulgare***  Chromosome Gene Name | | ***Zea mays*** |  |
|  |  | Chromosome | Gene Name |
| Hvchr1H | HORVU1Hr1G052560 | Zm09 | Zm00001eb393900 |
| Hvchr1H | HORVU1Hr1G083710 | Zm08 | Zm00001eb342960 |
| Hvchr2H | HORVU2Hr1G083090 | Zm10 | Zm00001eb425020 |
| Hvchr2H | HORVU2Hr1G091010 | Zm10 | Zm00001eb427140 |
| Hvchr2H | HORVU2Hr1G091010 | Zm02 | Zm00001eb075850 |
| Hvchr2H | HORVU2Hr1G091010 | Zm04 | Zm00001eb185690 |
| Hvchr2H | HORVU2Hr1G091010 | Zm05 | Zm00001eb248440 |
| Hvchr2H | HORVU2Hr1G103700 | Zm10 | Zm00001eb431140 |
| Hvchr3H | HORVU3Hr1G019880 | Zm03 | Zm00001eb121510 |
| Hvchr3H | HORVU3Hr1G020220 | Zm03 | Zm00001eb121640 |
| Hvchr3H | HORVU3Hr1G027730 | Zm03 | Zm00001eb119930 |
| Hvchr3H | HORVU3Hr1G027730 | Zm08 | Zm00001eb338610 |
| Hvchr3H | HORVU3Hr1G031720 | Zm03 | Zm00001eb119190 |
| Hvchr3H | HORVU3Hr1G055410 | Zm03 | Zm00001eb159590 |
| Hvchr3H | HORVU3Hr1G066930 | Zm03 | Zm00001eb155150 |
| Hvchr3H | HORVU3Hr1G066930 | Zm08 | Zm00001eb361300 |
| Hvchr3H | HORVU3Hr1G098020 | Zm03 | Zm00001eb142210 |
| Hvchr3H | HORVU3Hr1G098020 | Zm08 | Zm00001eb363500 |
| Hvchr4H | HORVU4Hr1G001430 | Zm07 | Zm00001eb307860 |
| Hvchr4H | HORVU4Hr1G009500 | Zm01 | Zm00001eb055840 |
| Hvchr4H | HORVU4Hr1G010910 | Zm01 | Zm00001eb056350 |
| Hvchr4H | HORVU4Hr1G010910 | Zm05 | Zm00001eb215430 |
| Hvchr4H | HORVU4Hr1G019090 | Zm02 | Zm00001eb091540 |
| Hvchr4H | HORVU4Hr1G019090 | Zm03 | Zm00001eb139010 |
| Hvchr4H | HORVU4Hr1G033170 | Zm04 | Zm00001eb165440 |
| Hvchr4H | HORVU4Hr1G069350 | Zm01 | Zm00001eb007220 |
| Hvchr4H | HORVU4Hr1G084710 | Zm01 | Zm00001eb001710 |
| Hvchr4H | HORVU4Hr1G084710 | Zm09 | Zm00001eb403730 |
| Hvchr4H | HORVU4Hr1G086540 | Zm01 | Zm00001eb001040 |
| Hvchr5H | HORVU5Hr1G017260 | Zm01 | Zm00001eb051240 |
| Hvchr5H | HORVU5Hr1G017260 | Zm01 | Zm00001eb030650 |
| Hvchr5H | HORVU5Hr1G017260 | Zm03 | Zm00001eb135250 |
| Hvchr5H | HORVU5Hr1G093830 | Zm01 | Zm00001eb056560 |
| Hvchr5H | HORVU5Hr1G093830 | Zm01 | Zm00001eb051240 |
| Hvchr5H | HORVU5Hr1G093830 | Zm03 | Zm00001eb135250 |
| Hvchr5H | HORVU5Hr1G093830 | Zm05 | Zm00001eb215260 |
| Hvchr5H | HORVU5Hr1G094580 | Zm05 | Zm00001eb215000 |
| Hvchr5H | HORVU5Hr1G106990 | Zm07 | Zm00001eb302280 |
| Hvchr7H | HORVU7Hr1G019930 | Zm09 | Zm00001eb377270 |
| Hvchr7H | HORVU7Hr1G027670 | Zm06 | Zm00001eb281660 |
| Hvchr7H | HORVU7Hr1G027670 | Zm09 | Zm00001eb375380 |
| Hvchr7H | HORVU7Hr1G039810 | Zm05 | Zm00001eb254150 |
| Hvchr7H | HORVU7Hr1G045450 | Zm05 | Zm00001eb253050 |
| Hvchr7H | HORVU7Hr1G045450 | Zm09 | Zm00001eb381220 |
| Hvchr7H | HORVU7Hr1G073410 | Zm10 | Zm00001eb416950 |
| Hvchr7H | HORVU7Hr1G073410 | Zm04 | Zm00001eb173090 |
| Hvchr7H | HORVU7Hr1G074240 | Zm04 | Zm00001eb173190 |
| Hvchr7H | HORVU7Hr1G074240 | Zm06 | Zm00001eb261740 |
| Hvchr7H | HORVU7Hr1G084630 | Zm06 | Zm00001eb276870 |
| Hvchr7H | HORVU7Hr1G084630 | Zm09 | Zm00001eb385880 |
| Hvchr7H | HORVU7Hr1G087940 | Zm09 | Zm00001eb384150 |
| Hvchr7H | HORVU7Hr1G111780 | Zm06 | Zm00001eb274960 |
| Hvchr7H | HORVU7Hr1G111780 | Zm09 | Zm00001eb389600 |
| Hvchr7H | HORVU7Hr1G113760 | Zm09 | Zm00001eb388650 |
| HvchrUn | HORVU0Hr1G008690 | Zm01 | Zm00001eb011010 |
| HvchrUn | HORVU0Hr1G008690 | Zm09 | Zm00001eb398420 |

| **Additional file 4: Table S4: Gene Ontology and Kyoto Encyclopedia of Genes and Genomes analysis of *HvWD40* genes.** | | | | | | | | | | | | | | | |
| --- | --- | --- | --- | --- | --- | --- | --- | --- | --- | --- | --- | --- | --- | --- | --- |
| Category | GOID | Description | | | | BgRatio | | pvalue | | padj | geneID | | | Count | |
| BP | GO:0006364 | rRNA processing | | | | 21/8527 | | 3.40E-09 | | 4.59E-08 | HvWD40-112/HvWD40-155/HvWD40-163/HvWD40-164 | | | | 4 |
| BP | GO:0016072 | rRNA metabolic process | | | | 21/8527 | | 3.40E-09 | | 4.59E-08 | HvWD40-112/HvWD40-155/HvWD40-163/HvWD40-164 | | | | 4 |
| BP | GO:0042254 | ribosome biogenesis | | | | 34/8527 | | 2.62E-08 | | 1.99E-07 | HvWD40-112/HvWD40-155/HvWD40-163/HvWD40-164 | | | | 4 |
| BP | GO:0022613 | ribonucleoprotein complex biogenesis | | | | 35/8527 | | 2.95E-08 | | 1.99E-07 | HvWD40-112/HvWD40-155/HvWD40-163/HvWD40-164 | | | | 4 |
| BP | GO:0034470 | ncRNA processing | | | | 58/8527 | | 2.37E-07 | | 1.28E-06 | HvWD40-112/HvWD40-155/HvWD40-163/HvWD40-164 | | | | 4 |
| BP | GO:0034660 | ncRNA metabolic process | | | | 110/8527 | | 3.14E-06 | | 1.41E-05 | HvWD40-112/HvWD40-155/HvWD40-163/HvWD40-164 | | | | 4 |
| BP | GO:0044085 | cellular component biogenesis | | | | 132/8527 | | 6.51E-06 | | 2.40E-05 | HvWD40-112/HvWD40-155/HvWD40-163/HvWD40-164 | | | | 4 |
| BP | GO:0006396 | RNA processing | | | | 135/8527 | | 7.12E-06 | | 2.40E-05 | HvWD40-112/HvWD40-155/HvWD40-163/HvWD40-164 | | | | 4 |
| BP | GO:0071840 | cellular component organization or biogenesis | | | | 270/8527 | | 0.00010926 | | 0.000327777 | HvWD40-112/HvWD40-155/HvWD40-163/HvWD40-164 | | | | 4 |
| BP | GO:0006886 | intracellular protein transport | | | | 80/8527 | | 0.00299858 | | 0.007773364 | HvWD40-69/HvWD40-134 | | | | 2 |
| BP | GO:0034613 | cellular protein localization | | | | 87/8527 | | 0.0035363 | | 0.007773364 | HvWD40-69/HvWD40-134 | | | | 2 |
| BP | GO:0070727 | cellular macromolecule localization | | | | 87/8527 | | 0.0035363 | | 0.007773364 | HvWD40-69/HvWD40-134 | | | | 2 |
| BP | GO:0046907 | intracellular transport | | | | 93/8527 | | 0.00403063 | | 0.007773364 | HvWD40-69/HvWD40-134 | | | | 2 |
| BP | GO:0051649 | establishment of localization in cell | | | | 93/8527 | | 0.00403063 | | 0.007773364 | HvWD40-69/HvWD40-134 | | | | 2 |
| BP | GO:0015031 | protein transport | | | | 106/8527 | | 0.00520596 | | 0.00823754 | HvWD40-69/HvWD40-134 | | | | 2 |
| BP | GO:0015833 | peptide transport | | | | 106/8527 | | 0.00520596 | | 0.00823754 | HvWD40-69/HvWD40-134 | | | | 2 |
| BP | GO:0042886 | amide transport | | | | 106/8527 | | 0.00520596 | | 0.00823754 | HvWD40-69/HvWD40-134 | | | | 2 |
| BP | GO:0045184 | establishment of protein localization | | | | 110/8527 | | 0.00559592 | | 0.00823754 | HvWD40-69/HvWD40-134 | | | | 2 |
| BP | GO:0008104 | protein localization | | | | 113/8527 | | 0.00589704 | | 0.00823754 | HvWD40-69/HvWD40-134 | | | | 2 |
| BP | GO:0051641 | cellular localization | | | | 115/8527 | | 0.00610188 | | 0.00823754 | HvWD40-69/HvWD40-134 | | | | 2 |
| BP | GO:0033036 | macromolecule localization | | | | 121/8527 | | 0.00673593 | | 0.008660487 | HvWD40-69/HvWD40-134 | | | | 2 |
| BP | GO:0016192 | vesicle-mediated transport | | | | 127/8527 | | 0.00739904 | | 0.009080643 | HvWD40-69/HvWD40-134 | | | | 2 |
| BP | GO:0071705 | nitrogen compound transport | | | | 146/8527 | | 0.00968708 | | 0.011371789 | HvWD40-69/HvWD40-134 | | | | 2 |
| BP | GO:0071702 | organic substance transport | | | | 151/8527 | | 0.01033589 | | 0.011627882 | HvWD40-69/HvWD40-134 | | | | 2 |
| BP | GO:0016567 | protein ubiquitination | | | | 53/8527 | | 0.05459413 | | 0.057736612 | HvWD40-52 | | | | 1 |
| BP | GO:0032446 | protein modification by small protein conjugation | | | | 54/8527 | | 0.05559822 | | 0.057736612 | HvWD40-52 | | | | 1 |
| BP | GO:0070647 | protein modification by small protein conjugation or removal | | | | 78/8527 | | 0.07941375 | | 0.079413746 | HvWD40-52 | | | | 1 |
| CC | GO:0030117 | membrane coat | | | | 23/2888 | | 0.00018118 | | 0.002717735 | HvWD40-69/HvWD40-134 | | | | 2 |
| CC | GO:0048475 | coated membrane | | | | 23/2888 | | 0.00018118 | | 0.002717735 | HvWD40-69/HvWD40-134 | | | | 2 |
| CC | GO:0098796 | membrane protein complex | | | | 176/2888 | | 0.01063681 | | 0.028817348 | HvWD40-69/HvWD40-134 | | | | 2 |
| CC | GO:0005798 | Golgi-associated vesicle | | | | 13/2888 | | 0.0134481 | | 0.028817348 | HvWD40-69 | | | | 1 |
| CC | GO:0012506 | vesicle membrane | | | | 13/2888 | | 0.0134481 | | 0.028817348 | HvWD40-69 | | | | 1 |
| CC | GO:0030120 | vesicle coat | | | | 13/2888 | | 0.0134481 | | 0.028817348 | HvWD40-69 | | | | 1 |
| CC | GO:0030135 | coated vesicle | | | | 13/2888 | | 0.0134481 | | 0.028817348 | HvWD40-69 | | | | 1 |
| CC | GO:0030659 | cytoplasmic vesicle membrane | | | | 13/2888 | | 0.0134481 | | 0.028817348 | HvWD40-69 | | | | 1 |
| CC | GO:0030660 | Golgi-associated vesicle membrane | | | | 13/2888 | | 0.0134481 | | 0.028817348 | HvWD40-69 | | | | 1 |
| CC | GO:0030662 | coated vesicle membrane | | | | 13/2888 | | 0.0134481 | | 0.028817348 | HvWD40-69 | | | | 1 |
| CC | GO:0031410 | cytoplasmic vesicle | | | | 13/2888 | | 0.0134481 | | 0.028817348 | HvWD40-69 | | | | 1 |
| CC | GO:0031982 | vesicle | | | | 13/2888 | | 0.0134481 | | 0.028817348 | HvWD40-69 | | | | 1 |
| CC | GO:0044433 | cytoplasmic vesicle part | | | | 13/2888 | | 0.0134481 | | 0.028817348 | HvWD40-69 | | | | 1 |
| CC | GO:0097708 | intracellular vesicle | | | | 13/2888 | | 0.0134481 | | 0.028817348 | HvWD40-69 | | | | 1 |
| CC | GO:0044422 | organelle part | | | | 238/2888 | | 0.01918919 | | 0.034398631 | HvWD40-69/HvWD40-155 | | | | 2 |
| CC | GO:0044446 | intracellular organelle part | | | | 238/2888 | | 0.01918919 | | 0.034398631 | HvWD40-69/HvWD40-155 | | | | 2 |
| CC | GO:0005794 | Golgi apparatus | | | | 20/2888 | | 0.02063918 | | 0.034398631 | HvWD40-69 | | | | 1 |
| CC | GO:0044431 | Golgi apparatus part | | | | 20/2888 | | 0.02063918 | | 0.034398631 | HvWD40-69 | | | | 1 |
| CC | GO:0098805 | whole membrane | | | | 31/2888 | | 0.03186871 | | 0.050319017 | HvWD40-69 | | | | 1 |
| CC | GO:0098588 | bounding membrane of organelle | | | | 39/2888 | | 0.0399815 | | 0.059972251 | HvWD40-69 | | | | 1 |
| CC | GO:0031090 | organelle membrane | | | | 57/2888 | | 0.0580693 | | 0.08295614 | HvWD40-69 | | | | 1 |
| CC | GO:0031981 | nuclear lumen | | | | 64/2888 | | 0.06504162 | | 0.088693113 | HvWD40-155 | | | | 1 |
| CC | GO:0012505 | endomembrane system | | | | 79/2888 | | 0.07986626 | | 0.094420337 | HvWD40-69 | | | | 1 |
| CC | GO:0031974 | membrane-enclosed lumen | | | 81/2888 | | | 0.08183096 | | 0.094420337 | HvWD40-155 | | | | 1 |
| CC | GO:0043233 | organelle lumen | | | 81/2888 | | | 0.08183096 | | 0.094420337 | HvWD40-155 | | | | 1 |
| CC | GO:0070013 | intracellular organelle lumen | | | 81/2888 | | | 0.08183096 | | 0.094420337 | HvWD40-155 | | | | 1 |
| CC | GO:0044428 | nuclear part | | | 96/2888 | | | 0.09647712 | | 0.107196801 | HvWD40-155 | | | | 1 |
| CC | GO:0043228 | non-membrane-bounded organelle | | | 439/2888 | | | 0.39033134 | | 0.40073164 | HvWD40-155 | | | | 1 |
| CC | GO:0043232 | intracellular non-membrane-bounded organelle | | | 439/2888 | | | 0.39033134 | | 0.40073164 | HvWD40-155 | | | | 1 |
| CC | GO:0005634 | nucleus | | | 453/2888 | | | 0.40073164 | | 0.40073164 | HvWD40-155 | | | | 1 |
| MF | GO:0004842 | ubiquitin-protein transferase activity | | | 61/13080 | | | 0.41168946 | | 0.617534184 | HvWD40-52 | | | | 1 |
| MF | GO:0019787 | ubiquitin-like protein transferase activity | | | 61/13080 | | | 0.41168946 | | 0.617534184 | HvWD40-52 | | | | 1 |
| MF | GO:0005198 | structural molecule activity | | | 380/13080 | | | 0.84462642 | | 0.844626423 | HvWD40-69/HvWD40-134 | | | | 2 |
|  | | | | | | | | | | | | | | | |
| KEGGID | Description | | BgRatio | pvalue | | | padj | | geneID | | | keggID | | Count | |
| ath03013 | RNA transport | | 138/3707 | 6.67E-06 | | | 6.67E-05 | | HvWD40-11/HvWD40-38/HvWD40-48/HvWD40-54/HvWD40-81/HvWD40-104/HvWD40-106/HvWD40-107 | | | | ath:AT1G15470/ath:AT3G01340/ath:AT2G30050/ath:AT1G64350/ath:AT2G46290/ath:AT3G56900/ath:AT2G19430/ath:AT3G15610 | 8 | |
| ath03008 | Ribosome biogenesis in eukaryotes | | 81/3707 | 0.00034612 | | | 0.00173061 | | HvWD40-18/HvWD40-71/HvWD40-89/HvWD40-54/HvWD40-155 | | | | ath:AT1G15440/ath:AT4G07410/ath:AT3G21540/ath:AT4G04940/ath:AT2G47990 | 5 | |
| ath03015 | mRNA surveillance pathway | | 103/3707 | 0.00104903 | | | 0.00349677 | | HvWD40-15/HvWD40-75/HvWD40-111/HvWD40-116/HvWD40-121 | | | | ath:AT5G14530/ath:AT5G66240/ath:AT5G13480/ath:AT5G60940/ath:AT1G17720 | 5 | |
| ath03040 | Spliceosome | | 156/3707 | 0.03155868 | | | 0.0788967 | | HvWD40-50/HvWD40-79/HvWD40-80/HvWD40-152 | | | | ath:AT4G15900/ath:AT2G41500/ath:AT1G10580/ath:AT2G43770 | 4 | |
| ath04120 | Ubiquitin mediated proteolysis | | 109/3707 | 0.05192385 | | | 0.10384769 | | HvWD40-59/HvWD40-64/HvWD40-90 | | | | ath:AT1G27840/ath:AT5G13840/ath:AT4G22910 | 3 | |
| ath03018 | RNA degradation | | 98/3707 | 0.17775765 | | | 0.29408481 | | HvWD40-78/HvWD40-127 | | | | ath:AT4G29830/ath:AT3G13300 | 2 | |
| ath04712 | Circadian rhythm - plant | | 31/3707 | 0.21687446 | | | 0.29408481 | | HvWD40-19 | | | | ath:AT2G46340 | 1 | |
| ath04136 | Autophagy-other | | 34/3707 | 0.23526785 | | | 0.29408481 | | HvWD40-67 | | | | ath:AT3G18140 | 1 | |
| ath04141 | Protein processing in endoplasmic reticulum | | 144/3707 | 0.31167639 | | | 0.3463071 | | HvWD40-38/HvWD40-48 | | | | ath:AT3G01340/ath:AT2G30050 | 2 | |
| ath03420 | Nucleotide excision repair | | 57/3707 | 0.36307042 | | | 0.36307042 | | HvWD40-59 | | | | ath:AT1G27840 | 1 | |

| **Additional file 5: Table S5: Experimental primers** | | |
| --- | --- | --- |
| Primer name | Primer sequence（5′→3′） | Purpose |
| HvnANT1 | F: GGGAGAAGAGCCACCATTTCA | qRT-PCR primer |
|  | R: CAGGGACTTGAGCCATTCCT |  |
| HvnANT2 | F: GAAGAAAGCTTTGGCCGGTG | qRT-PCR primer |
|  | R: TCCACCTTGTGAATGGACGG |  |
| HvnWD40-140 | F: GAAGAAAGCTTTGGCCGGTG | qRT-PCR primer |
|  | R: TCCACCTTGTGAATGGACGG |  |
| TC139057 | F: GAAGGATGAGCAAAAGGCCCT | Internal reference primer |
|  | R: GGCAGGCAGACTCATTTCTTCC |  |
| HvnANT1-GFP | F: GGGGACAAGTTTGTACAAAAAAGCAGGCTTCATGGGGAGGAGGGCGTGCTGT | Subcellular localization primer |
|  | R: GGGGACCACTTTGTACAAGAAAGCTGGGTCTTAACCGGCCATGTGCAGGGA |  |
| HvnANT2-GFP | F: GGGGACAAGTTTGTACAAAAAAGCAGGCTTCATGGTAATGGCGCTACCAAT | Subcellular localization primer |
|  | R: GGGGACCACTTTGTACAAGAAAGCTGGGTCTCAGCGCCCGCGTGTAG |  |
| HvnWD40-140-GFP | F: GGGGACAAGTTTGTACAAAAAAGCAGGCTTCATGGCGACGACGAGCGAGTCC | Subcellular localization primer |
|  | R: GGGGACCACTTTGTACAAGAAAGCTGGGTCTTATGTCCTGGGAACTGTAAC |  |
| HvnANT1-YC-YFP | F: CATTTACGAACGATAGTTAATTAAATGGGGAGGAGGGCG | BiFC primer |
|  | R: CACTGCCACCTCCTCCACTAGTACCGGCCATGTGCAG |  |
| HvnANT1-YN-YFP | F: CATTTACGAACGATAGTTAATTAAATGGGGAGGAGGGCG | BiFC primer |
|  | R: ACTGCCACCTCCTCCACTAGTACCGGCCATGTGCAG |  |
| HvnANT2-YC-YFP | F: CATTTACGAACGATAGTTAATTAAATGGTAATGGCGCTACCAAT | BiFC primer |
|  | R: CACTGCCACCTCCTCCACTAGTTCAGCGCCCGCGTGTAGCTG |  |
| HvnWD40-140-YN-YFP | F: CATTTACGAACGATAGTTAATTAAATGGCGACGACGAGC | BiFC primer |
|  | R: ACTGCCACCTCCTCCACTAGTTGTCCTGGGAACTGT |  |
| HvnANT1-BD | F: ATGGCCATGGAGGCCGAATTCATGGGGAGGAGGGCGTGC | Y2H primer |
|  | R: CCGCTGCAGGTCGACGGATCCTTAACCGGCCATGTGCAG |  |
| HvnANT1-AD | F: GCCATGGAGGCCAGTGAATTCATGGGGAGGAGGGCGTGC | Y2H primer |
|  | R: CAGCTCGAGCTCGATGGATCCTTAACCGGCCATGTGCAG |  |
| HvnANT2-BD | F: GCCATGGAGGCCAGTGAATTCATGGTAATGGCGCTACCAAT | Y2H primer |
|  | R: CAGCTCGAGCTCGATGGATCCTCAGCGCCCGCGTGTAGCTG |  |
| HvnWD40-140-AD | F: GCCATGGAGGCCAGTGAATTCATGGCGACGACGAGC | Y2H primer |
|  | R: CAGCTCGAGCTCGATGGATCCTTATGTCCTGGGAAC |  |
| HvnDFR-Pro-LUC | F: CGAGGTCGACGGTATCGATACAGCAGTACCAGAAGAGTTCTTTTACGG | Dual-Luciferase Reporter primer |
|  | R: CGAGGTCGACGGTATCGATACAGCAGTACCAGAAGAGTTCTTTTACGG |  |
| HvnANT1-LUC | F: GGAGAGGACAGCCCACCACCATGGCGACGACGAGCGAGTC | Dual-Luciferase Reporter primer |
|  | R: AGAGACTGGTGATTTCAGCGTTATGTCCTGGGAACTGTAACATTCAAGATCTTC |  |
| HvnANT2-LUC | F: GGAGAGGACAGCCCACCACCATGGGGAGGAGGGCGTGC | Dual-Luciferase Reporter primer |
|  | R: AGAGACTGGTGATTTCAGCGTTAACCGGCCATGTGCAGGGAC |  |
| HvnWD40-140-LUC | F: GGAGAGGACAGCCCACCACCATGGTAATGGCGCTACCAATAGTTCGTC | Dual-Luciferase Reporter primer |
|  | R: AGAGACTGGTGATTTCAGCGTCAGCGCCCGCGTGTAGC |  |

**
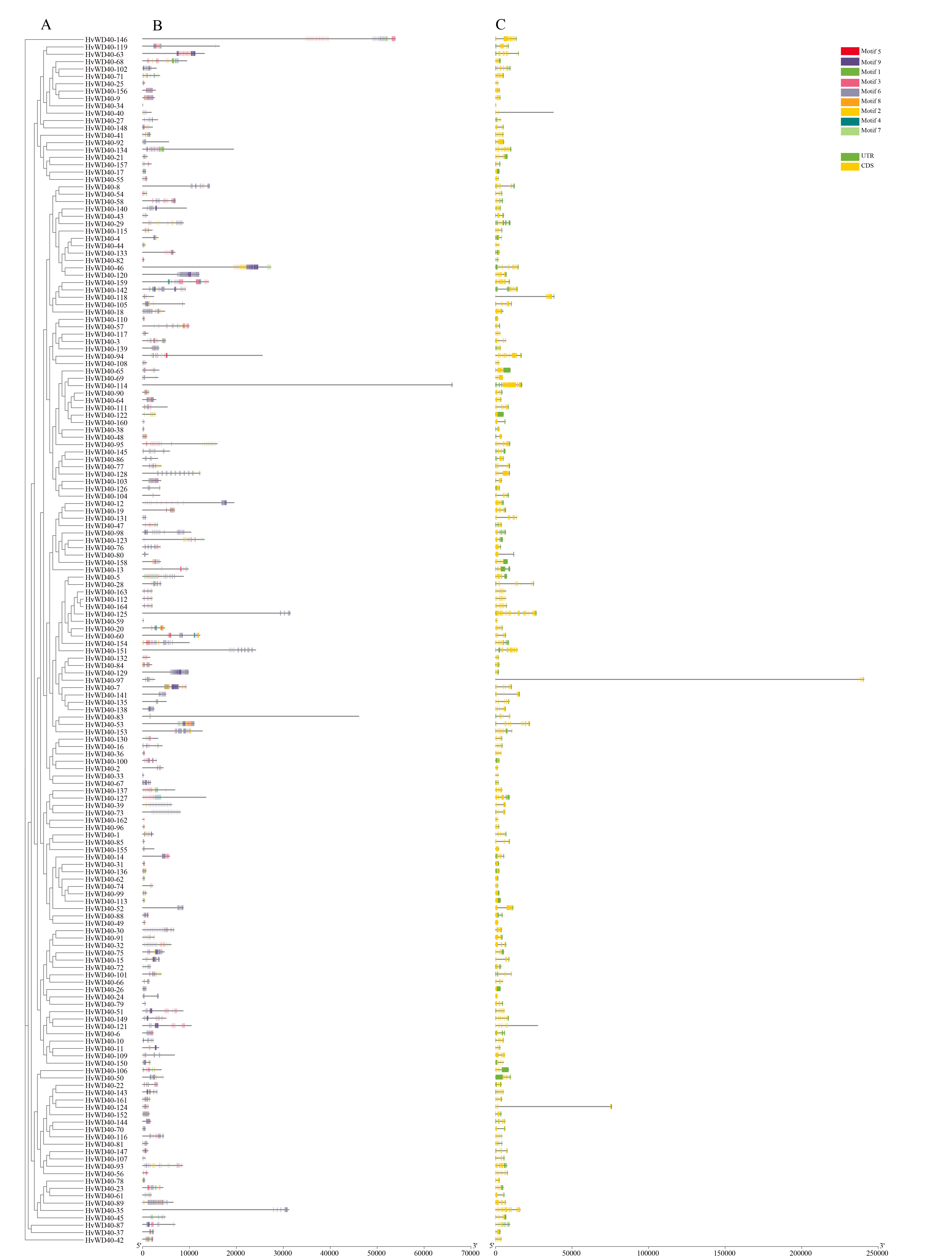
**

**Additional file 6: Fig. S1.** Phylogenetic relationships, gene structure, and architecture of conserved protein motifs in 164 HvWD40 from *Hordeum vulgare*. **A** Evolutionary tree analysis. **B** Motif composition of HvWD40 protein. The motif, numbers 1-9, is displayed in different colored boxes. **C** Exon, intron and UTR structure of 164 *HvWD40* genes. The green box shows the untranslated 5' and 3' regions, the yellow box shows the exons, and the black line shows the introns. Gene length and protein length can be estimated by the scale at the bottom.


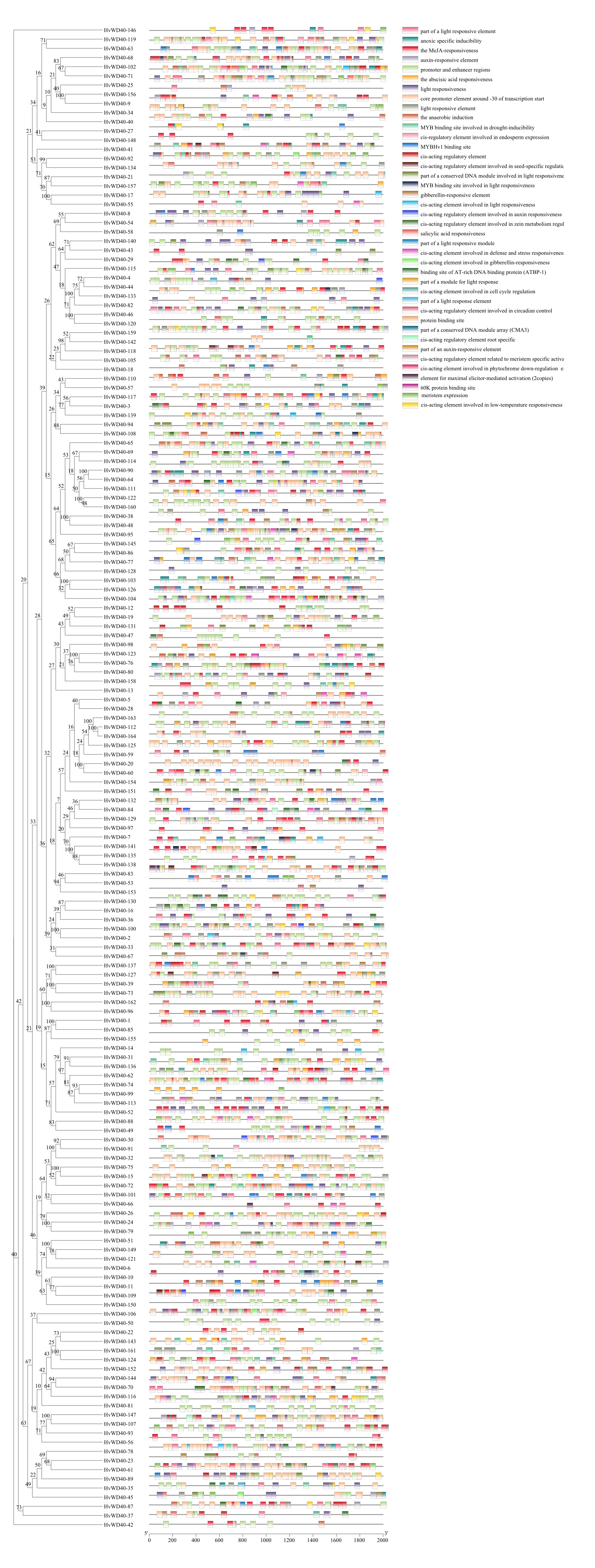


**Additional file 7: Fig. S2.** The information of cis-acting elements on the promoters of 164 *HvWD40* genes. Genes with promoter regions located at 2000 bp upstream of each *HvWD40* were evaluated by PlantCARE analysis. Different colors indicate different cis-acting elements.


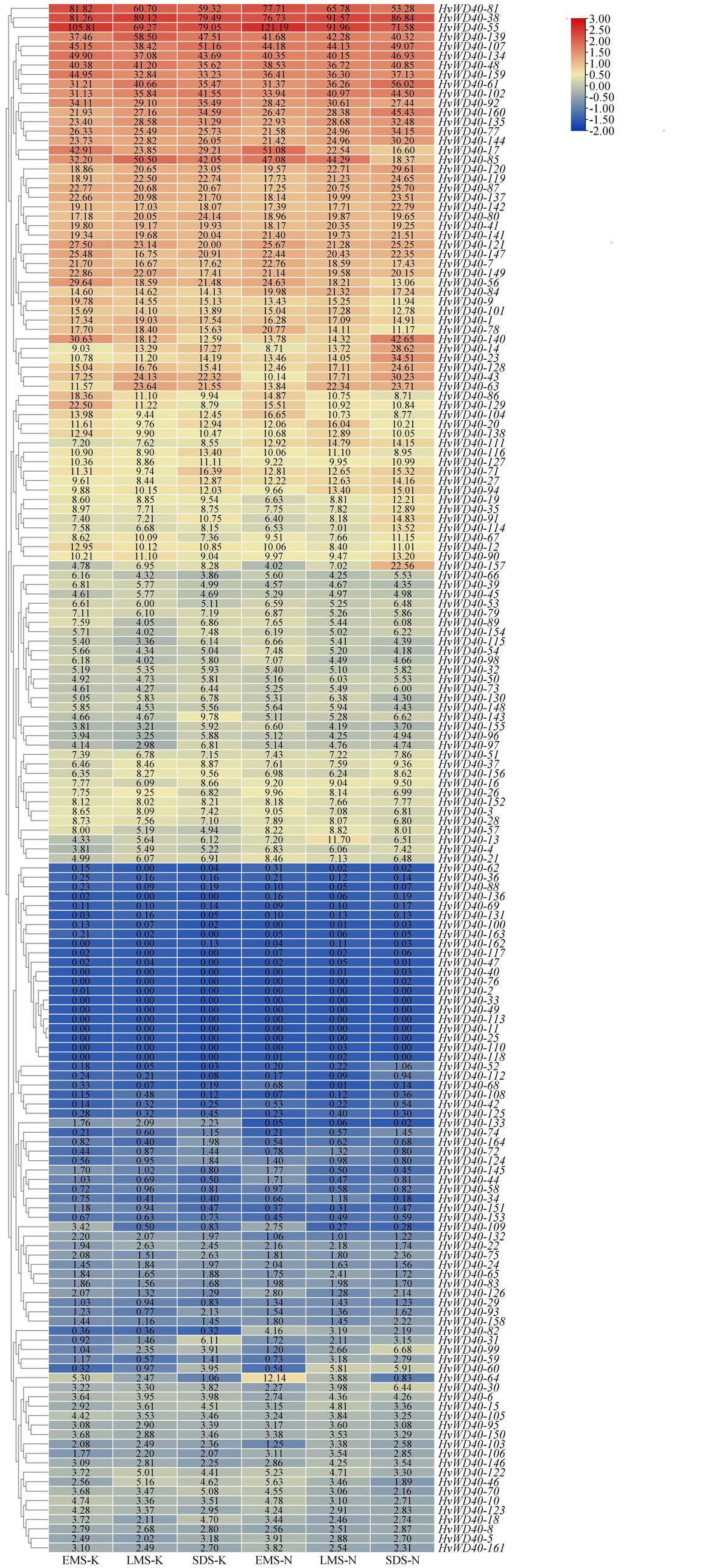


**Additional file 8: Fig. S3.** Expression profiling of 164 *HvWD40* genes. EMS-K: early mlik stage of Kunlun10; LMS-K: late milk stage of Kunlun10; SDS-K: soft dough stage of Kunlun 10; EMS-N: early milk stage of Nierumuzha; LMS-N: late milk stage of Nierumuzha; SDS-N: soft dough stage of Nierumuzha. The heatmap was generated on the basis of the RNA-seq data and drawn with the TBtools program. Different colors cell correspond to the log10 magnitude of the difference in expression level [log10 (fold change values+1)]. A redder color cell indicate upregulation, while bluer cell color indicate downregulation.
